# Acupuncture or auricular electro-acupuncture as adjuncts to lifestyle interventions for weight management in PCOS: protocol for a randomised controlled feasibility study

**DOI:** 10.1186/s40814-020-00591-4

**Published:** 2020-04-25

**Authors:** Carolyn Ee, Caroline A. Smith, Michael Costello, Lisa Moran, Genevieve Z. Steiner, Nigel Stepto, Adele Cave, Atekah Albrehee, Helena Teede

**Affiliations:** 1 NICM Health Research Institute, Western Sydney University, Penrith, NSW 2751 Australia; 2 Graduate Research School, Western Sydney University, Penrith, NSW 2751 Australia; 3 School of Women’s and Children’s Health, Level 1, Women’s Health Institute, Royal Hospital for Women, Randwick, NSW 2031 Australia; 4 Monash Centre for Health Research and Implementation, Locked Bag, Clayton, VIC 29 Australia; 5 Institute for Heath and Sport, Victoria University, Melbourne, VIC Australia

**Keywords:** Polycystic ovary syndrome, PCOS, Obesity, Weight management, Acupuncture, Auricular electro-acupuncture, Heart rate variability, Insulin resistance

## Abstract

**Background:**

Polycystic ovary syndrome (PCOS) is a prevalent women’s health condition with reproductive, metabolic, and psychological manifestations. Weight loss can improve these symptoms and is a key goal; however, many women find this difficult to achieve. Acupuncture is a Chinese medical treatment that involves insertion of very fine metal needles into specific areas of the body and has been shown to be efficacious for weight loss in non-PCOS populations. However, few studies have been conducted in women with PCOS. A variant of acupuncture, auricular electro-acupuncture (AEA), may have beneficial effects on sympathetic tone, which is associated with insulin resistance, obesity and PCOS.

**Methods:**

This prospective three-arm open label parallel randomised controlled trial will assess feasibility and acceptability of acupuncture and/or AEA for weight loss in women with PCOS. We will enrol 39 women from the community aged between 18 and 45 years, with physician diagnosis of PCOS according to the Rotterdam criteria: body mass index (BMI) between 25 and 40 kg/m^2^. Women will be randomly allocated to receive one of three treatments for 12 weeks duration: body electro-acupuncture + lifestyle interventions, AEA + lifestyle interventions, or lifestyle interventions alone. The lifestyle intervention in this study is telephone-based health coaching (between 4 and 13 phone calls, depending on individual need), provided by the Get Healthy Service. Primary outcomes of the study are feasibility and acceptability of trial methods as determined by recruitment and retention rates, adherence, acceptability, credibility, and safety. Secondary outcomes include anthropometric (body weight, BMI, waist and hip circumference), metabolic (glucose tolerance and insulin sensitivity obtained from a 2-h 75 g oral glucose tolerance test with area under the curve insulin calculated using the trapezoid rule), reproductive (androgen levels, menstrual cyclicity, clinical hyperandrogenism using the Ferriman-Gallwey scoring system), autonomic (heart rate variability, blood pressure), lifestyle (physical activity levels, diet quality, weight self-efficacy), quality of life, and psychological (depression and anxiety symptoms, internal health locus of control).

**Discussion:**

This study addresses the feasibility and acceptability of novel interventions to treat overweight/obesity in PCOS. Study findings have the potential to generate a new understanding of the role of acupuncture and auricular acupuncture in weight management.

**Trial registration:**

Australian New Zealand Clinical Trial Registry, 8/6/18 ACTRN12618000975291

## Background

Polycystic ovary syndrome (PCOS) affects up to 13% of women and can have significant reproductive, metabolic, and psychological manifestations [1, 2]. Women with PCOS are more likely to be obese/overweight than age-matched controls, and excess weight worsens the features of PCOS [3]. Weight loss is a key goal in PCOS, and lifestyle management techniques (diet, exercise, and/or behavioural interventions) targeted at weight loss are first-line approaches in overweight/obese women as even modest weight loss improves reproductive and metabolic outcomes [4]. Current evidence-based guidelines on PCOS recommend that obese women embark on 3–6 months of lifestyle management for weight loss prior to considering assisted reproductive technology (ART). However, adherence is generally low [5, 6], and achieving adequate weight loss remains a significant challenge [4, 7].

In non-PCOS populations, acupuncture, in particular auricular acupuncture and electro-acupuncture, is more efficacious than sham for reducing body mass index (BMI) (mean difference [MD] − 0.47 kg/m^2^) as well as body fat mass (MD − 0.66 kg), waist circumference (MD − 2.02 cm) and hip circumference (MD − 2.74 cm) [8]. These effects are mediated through multiple responses including appetite suppression [9, 10], modulation of leptin and ghrelin [11–13], and improved insulin sensitivity [14–19]. Further, acupuncture may alleviate co-morbid anxiety symptoms in people with obesity [11, 20, 21]. Effects also appear to be sustained after end of treatment [22]. Randomised controlled trials (RCTs) conducted in China on women with PCOS indicate that acupuncture and metformin is superior to metformin alone in women with PCOS for reducing BMI [23]. One study reported a mean difference of 0.97 kg/m^2^ (95% CI 1.51, 0.43) for acupuncture and metformin compared to metformin alone [24].

A range of factors may contribute to increased BMI in PCOS, including abnormalities in energy homeostasis [3]. Insulin resistance (IR) has been demonstrated to be increased in women with PCOS compared to BMI-matched controls and is exacerbated by increased BMI [25, 26]. Increased sympathetic tone is an associated factor of IR [27] and has been identified as a potential therapeutic target in PCOS [28]. In rats with steroid-induced PCOS, electro-acupuncture reduces ovarian sympathetic hyper-innervation, ameliorates IR, and improves oestrous cycling [29]. Electro-acupuncture reduced muscle sympathetic nerve activity in PCOS women in one trial [27]. Our qualitative work indicates high levels of acceptance of acupuncture as a possible adjunct to lifestyle interventions for weight loss [30].

A variant of acupuncture is auricular electro-acupuncture (AEA). Evidence suggests that AEA stimulates the auricular branch of the vagus nerve which increases parasympathetic tone [31], deactivates the sympathetic nervous system [31], and suppresses appetite [9].

Given that acupuncture is a relatively safe treatment [32–34] with preliminary evidence suggesting it may be beneficial for weight loss in PCOS when used as an adjunct, rigorous clinical research in this area is warranted. The primary objective of this study is to assess feasibility and acceptability of trial procedures for a randomised controlled trial comparing body acupuncture, AEA, and lifestyle interventions alone for weight loss in women with PCOS. Secondary objectives are to [1] determine an effect size for body electro-acupuncture or AEA and lifestyle interventions versus lifestyle interventions alone for change in body weight for limited efficacy testing and to inform future sample size calculations and [2] explore the impact of body electro-acupuncture and auricular electro-acupuncture on sympathetic tone and insulin resistance. The primary outcome for a subsequent phase IIb trial will be change in weight in kilogrammes.

## Methods/design

This is a prospective three-arm open-label parallel randomised controlled trial taking place over a 12-week intervention period. Ethics approval was obtained from the Human Research Ethics Committee, Western Sydney University, H11973 (27 February 2017). The trial was prospectively registered on the Australian New Zealand Clinical Trial Registry, ACTRN12618000975291, on 08 June 2018. The study protocol (version 12, 28 August 19) (see Additional file 1) has been designed according to the SPIRIT guidelines [35] and Good Clinical Practice guidelines. The trial commenced recruiting in April 2019 and enrolled the first participant on 27 May 2019.

### Recruitment, setting, and informed consent

Women living in Sydney, Australia, will be recruited via advertising through consumer organisations, social media, University staff and student networks, and fertility and specialty PCOS clinics. To reduce selection bias, we will attempt to recruit from a wide variety of sources targeting the general population. Screening according to eligibility criteria is initially done through an online survey and confirmed at the baseline visit where written informed consent will be provided. Participants attend for body acupuncture and AEA treatment at private acupuncture clinics.

### Eligibility criteria

The inclusion criteria are as follows: women who are aged between 18 and 45, with physician diagnosis of PCOS within the previous 5 years according to the 2003 Rotterdam Criteria [36]; BMI ≥ 25 kg/m^2^ and < 40kg/m^2^; no diagnosis of other endocrine disorders; not on the following medications in the 3 months preceding enrolment: metformin or other medications affecting insulin and glucose metabolism, hormonal contraceptives or hormonal treatments for PCOS/assisted reproductive techniques including gonadotropins and the oral contraceptive pill or hormonal intrauterine device, pharmaceutical or complementary (including nutritional/herbal) treatments for weight loss.

Exclusion criteria include:

- Planning to conceive within the next 3 months;
- Not willing to avoid pregnancy for the duration of the study;
- Currently pregnant or breastfeeding;
- Less than 6 weeks postpartum;
- Breastfeeding within the previous 6 weeks;
- Needle acupuncture in the previous 3 weeks;
- Unable or unwilling to provide informed consent;
- Anticoagulant use;
- Pacemaker use;
- Immunocompromise; or
- Valvular heart disease.

### Randomisation, allocation concealment, and blinding

Women are randomised in a 1:1:1 ratio to receive either body acupuncture and lifestyle intervention, AEA and lifestyle intervention, or lifestyle intervention alone. Randomisation was performed in permuted blocks. The randomisation sequence was created using a computer programme (www.sealedenvelope.com) by a researcher external to the research team. This researcher will hold the randomisation sequence and created a series of 39 consecutively numbered sealed opaque envelopes that contain the ID number and allocation. The research assistant allocates participants by selecting the next consecutively number sealed opaque envelope that contains the allocation. Once the participant has had eligibility confirmed and has provided written informed consent, she is enrolled and given the next consecutive randomisation ID number, and the research assistant will open the numbered envelope to reveal the allocation. Before opening the envelope, the research assistant writes the participant’s name on the envelope and the date and signs the envelope as a record of randomisation. Investigators (outcome assessors and investigators involved in statistical analysis) are blinded to treatment allocation, treating acupuncturists and participants are not.

### Treatment schedule

#### Body acupuncture

Chinese medicine needle acupuncture will be delivered in a semi-pragmatic setting, for a total of 11 treatments of acupuncture over 12 weeks (twice a week for the first 2 weeks, weekly for 4 weeks, then fortnightly for 3 treatments). A minimum of six study acupuncturists, located across different areas of metropolitan Sydney, have been chosen to administer the treatments. They have a bachelor’s degree in Chinese Medicine, have five or more years of clinical experience, and are registered as Chinese medicine practitioners (acupuncture) with the Australian Health Practitioner Regulation Agency (AHPRA). Study acupuncturists receive hands-on training from CE, an experienced acupuncture researcher, and registered acupuncturist. Refresher training is provided if there has been a break of 2 months or more in between administering the trial interventions to participants, and training is complemented by a detailed practitioner manual.

Acupuncturists are to perform Chinese medicine diagnosis based on history and examination as per usual clinical practice. The acupuncture point protocol is a semi-fixed protocol which delivers a traditionally-based style of acupuncture where treatment is designed on the basis of a traditional Chinese Medicine clinical assessment. In choosing the acupuncture prescription, study acupuncturists are given the following instructions:

- Treatment of obesity/overweight is the primary objective of the acupuncture treatment
- At least six acupuncture points must be chosen from a list of 17 core acupuncture points that will be provided to the practitioner. These points are chosen from the traditional points that are recommended for treatment of syndromes that are related to obesity and PCOS (based on literature and textbook review) and from the points used in previous acupuncture for obesity and PCOS trials [31, 37].
- Points on the chest or back must be avoided to avoid risk of pneumothorax.

A minimum of ten acupuncture needles will be inserted per session. The core acupuncture points to be used are ST36, SP6, ST25, ST40, CV12, CV6, LI11, SP9, CV9, CV3, CV4, LI4, KI13, KI7, ST28, LR3, and GB34. Table 1 outlines the location of the points and the Chinese medicine rationale for use of the point [38]. The use of a manualised semi-fixed protocol, where acupuncturists choose from a core group of acupuncture points according to the Chinese medicine diagnosis and follow a written protocol, ensures that our treatment protocol maintains fidelity to acupuncture as a medical system (which requires individualisation and flexibility) while standardising the treatment that is provided to conform to scientific research methodology [39, 40].

**Table 1:** Acupuncture point selection rationale

Acupuncture point	Relevant Chinese medicine indication(s) for PCOS	Location
Zu San Li (ST36)	Harmonises Stomach, fortifies Spleen and resolves damp, supports correct *Qi* original *Qi* and fosters tonifies *Qi* and nourishes Blood and *Yin*	Below the knee, 3 *cun*^a^ inferior to Dubi, one finger breadth lateral to the anterior crest of the tibia
San Yin Jiao (SP6)	Tonifies Spleen and Stomach *Qi* and resolves damp, harmonises Liver and tonifies Kidney regulates menstruation, harmonises Lower *Jiao*, invigorates Blood, calms the spirit	On the medial side of the lower leg, 3 *cun* superior to the prominence of the medial malleolus, in the depression close to the medial crest of the tibia
Tian Shu (ST25)	Regulates Intestines, Spleen and Stomach, resolves damp and damp heat, regulates *Qi* and Blood, eliminates stagnation	On the abdomen to 2 *cun* lateral to the umbilicus
Feng Long (ST40)	Transforms phlegm and dampness, clears phlegm from the heart and calms the spirit, activates the channel and alleviates pain	On the lower leg, midway between the tibiofemoral joint line (level with the popliteal crease) and the lateral malleolus, two finger- breadth later to Tiaokou ST38
Zhong Wan (CV12)	Harmonises the middle *Jiao*, tonifies the Stomach and fortifies the Spleen regulates *Qi*	On the midline of the abdomen, 4 *cun* above the umbilicus and midway between the umbilicus and the sternocostal angle.
Qi Hai (CV6)	Fosters original *Qi*, tonifies *Qi*, tonifies the Kidneys and fortifies *Yang*, rescues the collapse of *Yang*, regulates *Qi* and harmonies Blood	On the midline of abdomen, 1.5 *cun* inferior to the umbilicus and 3.5* cun* superior to pubic symphysis
Qu Chi (LI11)	Clears heat, cools Blood, eliminates wind, drains damp and alleviates itching, regulates *Qi* and Blood	At the elbow, midway between Chize LU5 and the lateral epicondyle of the humerus, at the lateral end of the transverse cubital crease. (This point should be located with a flexed elbow.)
Yin Ling Quan (SP9)	Regulates Spleen and resolves damp, opens and moves water passages and benefits the lower *Jiao*	On the medial side of the leg, in the depression in the angle formed by the media condyle of the of the tibia and the posterior border of the tibia
Shui Fen (CV9)	Regulates the water passages, harmonises the intestines and dispels accumulations	On the midline of the abdomen, 1 *cun* above the umbilicus and 7 *cun* below the sternocostal angle
Zhong Ji (CV3)	Regulates *Qi* transformation and drains damp heat, drain dampness, benefits the uterus and regulates menstruation, dispels stagnation and benefits the lower *Jiao*, fortifies the Kidney	On the midline of the lower abdomen, 4 *cun* inferior to the umbilicus and 1 *cun* superior to the pubic symphysis
Guan Yuan (CV4)	Fortifies original *Qi* and benefits essence, tonifies and nourishes the Kidney, warms and fortifies the Spleen, benefits the uterus and assists conception, regulates the lower *Jiao*	On the midline of the lower abdomen, 3 *cun* inferior to the umbilicus and 2 *cun* superior to the public symphysis
He Gu (LI4)	Activates the channel and restores the *Yang*	On the dorsum of the hand, between the first and second metacarpal bones, at the midpoint of the second metacarpal bone and close to its radial border
Qi Xue (KI13)	Regulates the penetrating and conception vessel, regulates the lower *Jiao*	On the lower abdomen, 3 *cun* below the umbilicus, 2 *cun* superior to the superior border of the symphysis pubis, 0.4 *cun* lateral to midline (Guanyuan CV4)
Fu Liu (KI7)	Benefits the Kidneys, regulates the water passages, drains damp and clears damp heat	On the medial aspect of the lower leg, in the depression 2 *cun* superior to Taixi KI3, on the anterior border of the Achilles tendon. (KI3 is located in the depression between the tip of the medial malleolus and the Achilles tendon)
Shui Dao (ST28)	Warms the lower *Jiao,* regulates menstruation and benefits the genital region	On the lower abdomen, 2 *cun* lateral to the midline and 3 *cun* inferior to the umbilicus level with Guanyuan Ren 4. Note: The 2 *cun* line located between the midline and palpable border of the rectus abdominis of the umbilicus.
Tai Chong (LR3)	Spreads Liver *Qi*, subdues Liver *Yang* and extinguishes wind, nourishes Liver Blood and Liver *Yin*, regulates menstruation, regulates lower *Jiao*	On the dorsum of the foot, in the hollow distal to the junction of the first and second metatarsal bones
Yang Ling Quan (GB34)	Spreads Liver *Qi* and benefits the lateral costal region, clears Liver and Gall Bladder damp heat	Below the later aspect of the knee, in the tender depression approx. 1 *cun* anterior and 1 *cun* inferior to the head of the fibula

Acupuncture point nomenclature as per World Health Organisation guidelines for meridian alphabetic codes

^a^A *cun* is a measurement used in locating acupoints and corresponds to the distance between the two medial ends of the creases of the interphalangeal joints, when the patient’s middle finger is flexed.

Acupuncture points will be generally needled bilaterally. A combination of manual and electrical stimulation (electro-acupuncture) will be used. *De qi*, or needle sensation, is widely considered to be an essential component of acupuncture treatment [41] and is defined as *numbness*, *heaviness*, *pressure*, *soreness*, *or tingling. De qi* will be obtained for each point using thrusting, twirling, and rotating, until the participant reports numbness, heaviness, pressure, soreness, or tingling, and needles are manipulated every 10 min to obtain *de qi.* Electro-acupuncture will be applied to ST 28 and SP 6 acupuncture points bilaterally (located on the lower abdomen and lower leg) and delivered using Therapeutics Goods Administration (TGA)-approved electro-acupuncture devices at low frequency (2 Hz) electrical pulses, 0.3 ms pulse width [42, 43], and continuous stimulation to the attached needles. The intensity of electro-acupuncture will be increased gently during each session until muscle contractions are felt or seen, but below the threshold of any discomfort or pain. Needles are retained for 30 min. Acupuncture needles are standard, single use, stainless steel needles, 0.20 × 30 mm. Practitioners will record details of each diagnosis and treatment, on an individual case report form which will be kept in a locked cupboard in the clinic and returned to the research team at the end of the study.

#### Auricular electro-acupuncture stimulation

**Fig. 1:**
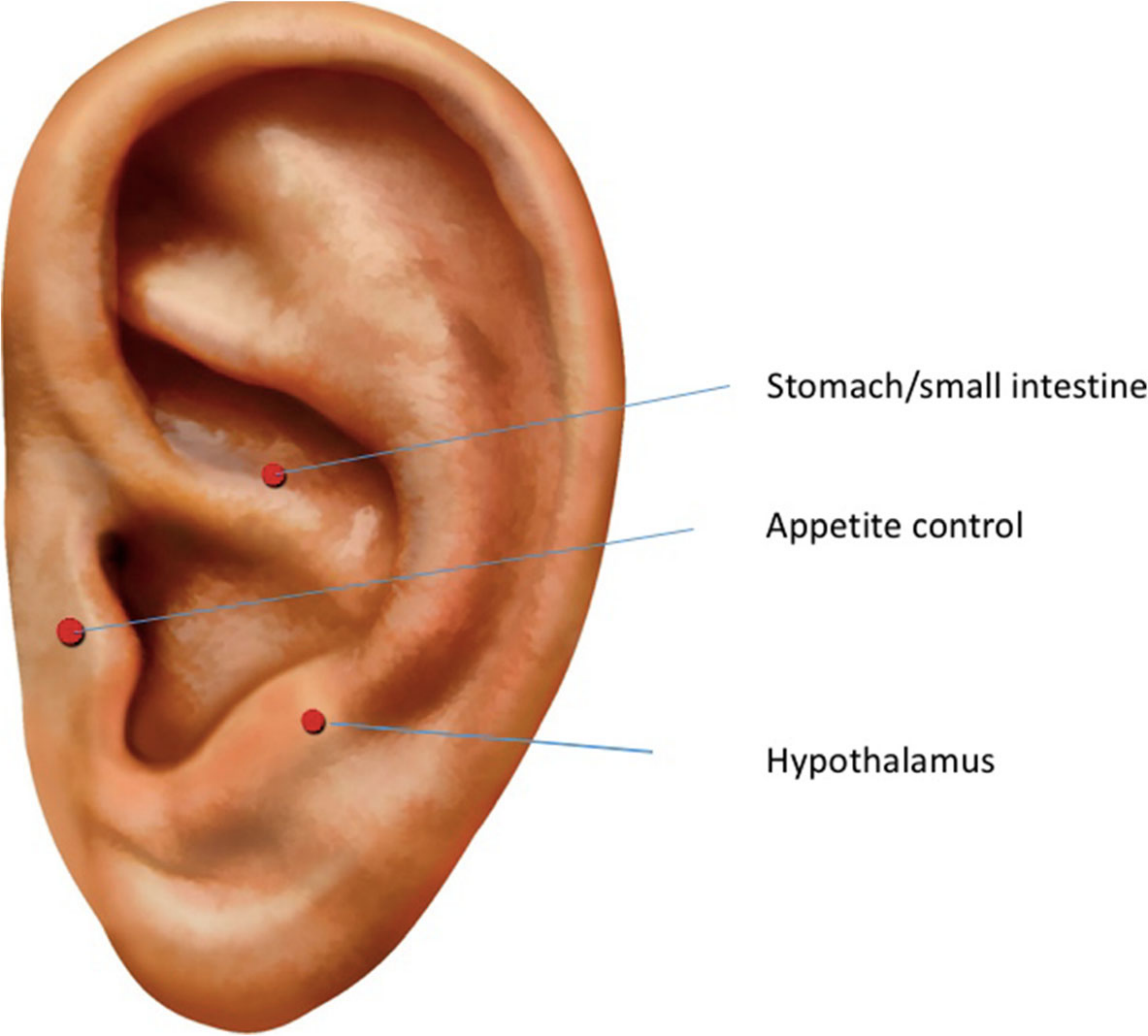
Location of ear acupuncture points

The intervention in this arm is AEA using a fixed protocol that is informed by both Chinese medical and medical acupuncture principles based on a literature review and expert consensus on the traditional and physiological indications of the points. A pilot study suggests that a novel device, the “Neurova^TM^” (NESI Corp), may be effective for hot flushes from androgen-deprivation therapy, a condition underpinned by sympathetic hyperactivity [44]. This method involves insertion of indwelling needles into the concha of the ear and delivery of intermittent electrical impulses over 96 h by a small battery-powered device that is worn just below the ear. This significantly increases the “dose” of acupuncture that can be delivered (“protracted acupuncture”). Women allocated to the AEA group will receive a total of 6 treatments over 12 weeks (once a week for the first 2 weeks, then fortnightly for four treatments), delivered by study acupuncturists. No additional Chinese medicine treatments will be provided including body acupuncture, Chinese herbal medicine, or additional physical or stimulation treatments such as moxibustion or cupping, and no Chinese medicine diagnosis is required. There is to be no variation in treatment. Each subject will receive needling at three acupuncture points on the concha of one ear only (stomach, appetite control, and thalamus) (see Fig. 1).

No *de qi* sensation is to be sought. The Neurova^TM^ device will be attached after insertion of the needles to the ear, according to the manufacturer’s instructions. Needles are retained for up to 96 h. The participant is to remove the Neurova^TM^ device by gently pulling out the needles and disconnecting the wire and discarding the device into an approved sharps disposal container. Participants can remove the device before 96 h if they experience significant discomfort. Needles are surgical-grade titanium, sterile and disposable, and are approximately 6 mm in length.

#### Lifestyle interventions only

The Get Healthy Service (GHS) is a free, confidential telephone-based coaching service that was introduced by New South Wales (NSW) Ministry of Health in 2009 [45]. The service is delivered by university-qualified health coaches, including dietitians and exercise physiologists, and is available to all residents of NSW aged 16 years and above. Health coaches are trained in HealthChange® Methodology which draws on principles and techniques similar to those used in motivational interviewing, solution-focused coaching, and cognitive behavioural therapy and provides an overarching framework for integration of these approaches into specific health promotion programmes. HealthChange® Methodology also integrates numerous models from evidence-based health behaviour change literature [46]. All participants will be referred to the GHS, and one group will be allocated to receive only the GHS. The intervention consists of a minimum of four and up to 13 individually tailored telephone calls exploring healthy eating, physical activity, and achieving and maintaining a healthy weight consistent with national guidelines, which are also recommended by international evidence-based PCOS guidelines [4, 47]. The frequency of health coaching is tailored according to individual need, coach and participant availability, and stage of programme, with more frequent calls generally required in the early stages.

#### Permitted and prohibited components of treatment

Women in the body acupuncture group may be provided diet and lifestyle advice according to Chinese medicine principles by the study acupuncturists. This advice usually consists of recommendations to avoid ingesting foods that are considered too “hot” or “cold” for the patient’s constitution. However, no additional Chinese medicine treatments will be provided including Chinese herbal medicine or additional physical or stimulation treatments such as moxibustion or cupping. Participants will be discouraged from using other co-interventions during the study. Any co-interventions used are recorded at the final study visit.

### Outcome measures

The primary outcomes for this study are feasibility and acceptability of recruitment methods, intervention, and outcome collection and will include:

- Recruitment rates (number of enquiries and number of enrolments per month of active recruitment; percentage conversion to enrolment measured as *n* enrolled/*n* of enquiries, and *n* enrolled/*n* potentially eligible);
- Retention rate (*n* completing 12-week intervention and outcome measures/*n* enrolled)
- Adherence rates (*n* completing at least 8 of 11 acupuncture treatments and 4 of 6 Neurova^TM^ treatments; *n* of hours of wear of the Neurova^TM^ device/percentage of women who wore the device for the prescribed 96 h duration; *n* completing all recommended phone calls with the GHS);
- Acceptability is measured using an exit survey which includes questions on perceived benefits of body acupuncture, AEA, and health coaching, perceived detriments of being in the trial, likelihood of recommending the study to family or friends measured on a 5 point Likert scale ranging from “I would definitely participate/recommend it” to “I would definitely not participate/recommend it”;
- Credibility of the treatment and expectancy related to treatment are collected immediately after the first acupuncture or AEA treatment, using a modified Credibility and Expectancy questionnaire [48]. To maintain blinding during data analysis, these data will be analysed after all other data have been interpreted.
- Safety (adverse events) which will be collected by study acupuncturists at each treatment, or recorded by the participant or research team at any time during the intervention period, using an Adverse Events Form

We will collect demographic and medical details using a survey at baseline, including age, ethnic background, smoking status, diet history, physical activity levels (number of 20-min sessions of vigorous and less vigorous exercise per week which will be converted to metabolic equivalents), family history of type 2 diabetes, acupuncture experience, and previous weight loss attempts. Concomitant medications are recorded at the baseline and 12-week visits.

The primary outcome for the phase IIb trial will be change in weight in kilogrammes from baseline to end of treatment (12 weeks). Secondary outcome measures include other anthropometric measures, metabolic outcomes, reproductive, psychological, quality of life, and lifestyle habits. These are collected at baseline and at end of treatment (12 weeks) and include the following:

#### Anthropometric measures

We will measure weight on a calibrated medical-grade digital scale (Omron) with the participant in light clothing and no shoes. We will measure waist and hip circumference using medical-grade tape and height by stadiometer at the baseline clinic visit, following procedures outlined in the National Health and Nutrition Examination Survey (NHANES) Anthropometry Procedures Manual [49].

#### Metabolic outcomes

Glucose tolerance and insulin sensitivity are obtained from a 2-h 75 g oral glucose and tolerance test collected by Laverty Pathology after an overnight fast. Serum is analysed for glucose levels using the hexokinase enzymatic method (ADVIA Chemistry Glucose Hexokinase_3 Concentrated Reagents (GLUH_c) enzymatic method) and for insulin using two-site sandwich immunoassay (ADVIA Centaur^R^ Insulin assay, ADVIA^R^ Chemistry Systems, Siemens Healthcare Diagnostics, Camberley, UK). Area under the curve (AUC) insulin will be calculated using the trapezoid method.

#### Reproductive outcomes

Menstrual cyclicity will be calculated from menstrual diaries over 12 weeks. Sex hormone-binding globulin, free testosterone and Free Androgen Index will be collected by Laverty Pathology and analysed using the ADVIA Centaur^R^ SHBG and Centaur^R^ Testosterone II (TSTII) assays. Clinical hyperandrogenism is calculated using the Ferriman-Gallwey scoring system [50].

#### Quality of life outcomes

We will assess both health-related and global quality of life using the validated Modified PCOS Questionnaire (MPCOSQ) [51] and EuroQol 5D (EQ-5D) questionnaire [52] respectively.

#### Psychological outcomes

We will assess depression and anxiety symptoms using the validated Depression Anxiety Stress Scale (DASS) 21 [53]. The construct of locus of control refers to the extent to which an individual believes that his or her environment and choices are under his or her control. This is relevant in obesity where an external locus of control, or the belief that events or outcomes are controlled by forces external to oneself, is correlated with greater adiposity [54]. We will measure locus of control using the validated Multidimensional Health Locus of Control questionnaire [55].

#### Lifestyle habits

- Physical activity: Participants will wear an accelerometer (Actigraph wGT3X+, initialised at 70 Hz) around the waist for 5–7 days (that includes 2 weekend days) and complete an associated sleep diary in the week before commencing the intervention, and in week 11
- Low eating self-efficacy, or poor confidence in one’s ability to control eating behaviour in the presence of challenging situations, has been identified as a potential barrier to long-term adherence to reduced energy intake. We will measure weight self-efficacy using the validated Weight Efficacy Lifestyle Questionnaire short form [56]
- Change in dietary habits (*n* of daily serves of vegetables and fruit, *n* of daily cups of sugar sweetened drinks, frequency of takeaway meals or snacks) is provided by the GHS at completion of the study

#### Autonomic outcomes

- Heart rate variability (HRV) provides non-invasive measures of both parasympathetic (high frequency/HF component) and sympathetic tone (low frequency/LF component) [57]. HRV will be calculated from electrocardiogram (ECG) recordings during the clinic visits. ECG recordings take place in the NICM Neurocognition and HEADBOX Labs at Western Sydney University. Women will be asked to avoid caffeine and smoking for at least 2 h prior to HRV measurement. Five minutes of seated eyes-closed resting-state 2-lead ECG will be recorded with pre-jelled disposable bipolar Ag/AgCl electrodes placed over the mid-sternum (medial to the fourth intercostal space; between V1 and V2) and fifth intercostal space at the left midaxillary line (V6) via a Compumedics Neuroscan Synamps2 Digital Signal Processing System and Neuroscan 4.5.1 Acquire software. Data will be sampled at 1000 Hz and amplified with the manufacturer’s default gain setting. HRV will be derived from ECG trace via MATLAB (Mathsworks®). HRV frequency bands will be defined as very low frequency (VLF; < 0.04 Hz), low frequency (LF; 0.04–0.15 Hz), and high frequency (HF; 0.18–0.40 Hz). LF/HF ratio will also be calculated as a measure of sympathovagal balance.
- Blood pressure is measured while sitting quietly and after resting quietly for a few minutes, and with both feet on the ground, using an Omron digital automatic standard blood pressure monitor. A large cuff is used if required. Blood pressure on both arms will be obtained initially and the higher blood pressure of the two will be used. Three measurements will be taken, and the average of the last two measurements is recorded. If there is > 10 mm/Hg or > 6mm/Hg difference in systolic or diastolic blood pressure respectively, the participant is asked to rest quietly for 5 min and blood pressure measurement is repeated [58].

### Criteria for success

We will consider this feasibility study a success if the following criteria are all met by the completion of all 12-week follow-up visits:

- The body acupuncture, AEA interventions, and lifestyle interventions are safe and acceptable to participants (no serious adverse events that are probably or definitely related to the interventions; at least 75% of women indicate that they would definitely or probably recommend participating in the trial to a friend with PCOS; at least 75% of women wore the Neurova^TM^ for more than 72 h at a time)
- Recruitment is feasible (recruitment target is reached; *n* of women enrolled per month of recruitment is at least 2; percentage of women enrolled compared to initial enquiry is greater than 12.5%; percentage of women enrolled compared to number of women who are potentially eligible is greater than 30%);
- Retention is satisfactory (percentage of women completing 12-week outcome measures compared to women enrolled is greater than 75%)
- Adherence is satisfactory in all three groups (percentage of women completing at least 66.67% of body acupuncture, AEA treatments or recommended telephone calls from GHS is greater than 75%)
- Mean credibility score is > 6 (credibility is measured on a 9-point Likert scale)

### Sample size

No sample size calculation is required as this is a feasibility study. We aim to analyse 30 participants by the end of treatment. Based on our collective experience, where trials which have required more intensive interventions have reported a dropout rate of over 40% in women with PCOS [59] while a randomised sham-controlled trial on acupuncture in menopausal women reported attrition of < 20% [60], we estimate a 25% dropout rate in this trial. Therefore, we aim to randomise 39 women (13 to body acupuncture, 13 to AEA, and 13 to lifestyle alone).

### Statistical analysis

Data relating to recruitment, retention, safety, adherence, acceptability, and credibility are presented with descriptive statistics (measures of central tendency, e.g., mean, variability; e.g., standard deviation; and effect size, e.g., 95% confidence intervals). Mixed model analysis of variance will be used to determine within-group differences for continuous outcomes between baseline and 12 weeks with time as a fixed effect and subject as a random effect and analysis of covariance for between-group differences for continuous outcomes with baseline score as a covariate, time and group as fixed effects, and subject as a random effect. Intention-to-treat analysis will be used.

### Quality assurance

Point location for body acupuncture and AEA and will be independently checked by an acupuncturist external to the study at least once for each practitioner. We will undertake regular calibration of digital weight scales and ECG equipment as per laboratory standard operating procedures.

### Safety monitoring

At each acupuncture treatment, study acupuncturists will enquire about adverse events (AE). All participants are provided with an AE form to complete if required and are provided with the principal investigator’s contact details to report all AE regardless of perceived causality. All AEs will be assessed for causality and severity by the chief investigator after consultation with other investigators (see Table 2) and followed up until resolution or until such time as the event is considered stable. Serious AE’s are reported to the Human Research Ethics Committee within 24 h of becoming known to the research team.

**Table 2:** Assessment of causality and severity of adverse events

Assessment of causality	Assessment of severity
**Unrelated**: where an event is not considered to be related to the study intervention	**Mild**: an event that is easily tolerated by the participant, causing minimal discomfort and not interfering with every day activities.
**Possibly**: although a relationship to the study intervention cannot be completely ruled out, the nature of the event, the underlying disease, concomitant medication or temporal relationship make other explanations possible.	**Moderate**: an event that is sufficiently discomforting to interfere with normal everyday activities.
**Probably**: the temporal relationship and absence of a more likely explanation suggest the event could be related to the study intervention.	**Severe**: an event that prevents normal everyday activities
**Definitely**: The known effects of the study intervention or its physiological mechanisms suggest that study intervention is the most likely cause.	

## Discussion

Obesity is a global health issue [61]. The worldwide prevalence of obesity has nearly tripled between 1975 and 2014 [62]. The obesity epidemic is a major contributor to the steep rise of type 2 diabetes, cancer, and cardiovascular disease (CVD) [63, 64]. The cost of obesity to the global health system is estimated at US$2 trillion annually [61]. Women with PCOS have a higher prevalence of being overweight or obese [3] including greater longitudinal weight gain [65]. Overweight and obesity worsens the features of PCOS [66], and weight management is recommended as initial first line treatment in international evidence-based guidelines in PCOS [4].

Women with PCOS express dissatisfaction with conventional medical treatments (such as fertility drugs and the oral contraceptive pill) and an overwhelming preference for alternatives that they perceive to be safer [67]. They are frequent users of complementary therapies, with up to a third using acupuncture [68]. Acupuncture may represent a low-risk [32] non-pharmacological adjunct to lifestyle interventions in women with PCOS and may contribute to reduction of the burden of chronic disease; however, there is a paucity of evidence from rigorous RCTs. Many RCTs fail due to inability to recruit to target [69]. To this end, the UK Medical Research Council recommends assessment of feasibility prior to full evaluation of complex interventions [70]. This can ensure that money spent on expensive trials is not wasted due to recruitment and retention failures.

This feasibility study will examine the safety, acceptability, and feasibility of recruitment and adherence to novel adjunctive interventions for weight loss in PCOS. Further, we will explore the impact of AEA, body acupuncture, and lifestyle interventions on measures of sympathetic tone and IR, and related biomarkers that are clinically relevant in PCOS, in order to build an accurate scientific argument for the biological plausibility of the interventions. Several sham-controlled RCTs on acupuncture for a variety of clinical conditions suggest that it is more efficacious than sham in reducing sympathetic tone as measured by HRV [57, 71, 72]. Previous clinical research showed that AEA resulted in improvement in HRV LF/HF ratios over the course of the protocol intervention, implying improved sympathovagal balance [44]; however, this was measured using ambulatory HRV monitoring, which has not been validated against the gold standard of electrocardiogram (ECG) recordings. To date, the effect of acupuncture on HRV in women with PCOS has not been evaluated, although electro-acupuncture improved HRV and restored oestrous cycling compared to sham handling in a recent rat study [73].

This study also provides vital real-world feasibility data on the acceptability of Neurova^TM^ device, which will allow for further modification of the device for improved health consumer comfort, convenience, and clinical effectiveness. This device represents an innovation in the delivery of acupuncture, allowing the device to be used at home, reducing the need for frequent clinic visits, and increasing the dose of acupuncture that can be delivered. Study findings will have the potential to generate new understandings of the role of acupuncture and allow greater exploration of the mechanisms underlying PCOS, a condition of significant health burden to women, with the potential to translate this research to other chronic conditions that are underpinned by autonomic dysfunction.

There are some limitations to the proposed research. It will not be possible to blind the intervention providers or the participants, therefore introducing the possibility of performance bias. To minimise this risk, we will conduct blinded outcome collection and analysis and utilise objective measures for outcomes wherever possible (such as accelerometers to measure physical activity levels). Further, due to operational and funding constraints, the study is limited to women in Sydney, and we will not be able to test the feasibility of a multicentre trial.

## Conclusion

This study addresses feasibility of a novel intervention to treat obesity, which is a global public health concern. With rising health costs and disease burden from obesity, there is an urgent need to identify effective treatments that are adjunctive to lifestyle interventions. Should the trial methods prove to be feasible and acceptable and meet the criteria for success, we will conduct a phase IIb randomised controlled trial assessing effectiveness and efficacy of the Neurova^TM^ device and body acupuncture as adjuncts to lifestyle interventions for obesity in women with PCOS.

## Supplementary information

**Additional file 1.** Acupuncture for PCOS protocol R1. SPIRIT FigureR1.

## Data Availability

Not applicable

## Abbreviations

AEA: Auricular electro-acupuncture
ART: Assisted reproductive technology
BMI: Body mass index
ECG: Electrocardiogram
GLUH_c: Glucose Hexokinase_3
GP: General practitioner
HRV: Heart rate variability
HF: High frequency
LF: Low frequency
IR: Insulin resistance
PCOS: Polycystic ovary syndrome
RCT: Randomised controlled trial
T2D: Type 2 diabetes
SHBG: Sex hormone-binding globulin
TGA: Therapeutic Goods Administration
TSTII: Testosterone II
WSU: Western Sydney University
