# Supplementary material for: Acupuncture or auricular electro-acupuncture as adjuncts to lifestyle interventions for weight management in PCOS: protocol for a randomised controlled feasibility study
Source: Pilot Feasibility Stud. 2020 Apr 25;6:53. doi: 10.1186/s40814-020-00591-4 (PMC7183107; doi:10.1186/s40814-020-00591-4)
Supplement: Supplementary file 1 — Additional file 1. Acupuncture for PCOS protocol R1. SPIRIT FigureR1. [file 40814_2020_591_MOESM1_ESM.zip › Appendix 1 Acupuncture for PCOS protocol R1.docx]

**CONFIDENTIAL**

**PROTOCOL TITLE**

**Acupuncture or auricular electro-acupuncture as adjuncts to lifestyle interventions for weight management in PCOS: a randomised controlled feasibility study**

**Protocol No: 012**

Date: 28/08/2019

**SPONSOR**

Western Sydney University through NICM Health Research Institute

Locked Bag 1797 Penrith

NSW 2751

This is an investigator initiated study led by Dr Ee who is a WSU employee. Dr Ee and the rest of the team have taken on responsibilities of sponsor and investigator roles. Dr Ee retains full control over study design, data collection, study management, analysis and interpretation of the data, writing of the report, and submitting the report for publication.

**SOURCES OF FUNDING**

NHMRC CRE in PCOS Project Grant 2017 (Competitive grant)

Partnership grant – Neural Ear Stimulation International (NESI) and NICM HRI (Non competitive grant – consultancy)

Chief Investigators of the CRE in PCOS are included as investigators in this study (CIs Teede and Moran). This was a criteria for grant eligibility. However, the CRE in PCOS in itself will not have any role in study design, data collection, study management, analysis and interpretation of the data, writing of the report, or submitting the report for publication apart from the involvement of the two CIs.

NESI provides advice on the use of the Neurova device, including suggested frequency of treatment, and acupuncture points to be used, but does not otherwise play any role in study design, data collection, study management, analysis and interpretation of the data, writing of the report, or submitting the report for publication

**TRIAL REGISTRATION**

Australian New Zealand Clinical Trial Registry, ACTRN12618000975291 08/06/2018

**PRINCIPAL CLINICAL INVESTIGATOR**

DR CAROLYN EE

RESEARCH FELLOW

NICM Health Research Institute

Western Sydney University Westmead Campus

158-160 Hawkesbury Road, Westmead

Locked Bag 1797 Penrith

NSW 2751

Phone: 02 46203085

PROF CAROLINE SMITH

Graduate Research School, Western Sydney University

NSW

**CHIEF INVESTIGATORS**

Dr Michael Costello

School of Women’s and Children’s Health

Level 1, Women’s Health Institute

Royal Hospital for Women

Randwick NSW 2031

A/Prof Lisa Moran

Monash Centre for Health Research and Implementation

Locked Bag 29

Clayton

Victoria

Prof Helena Teede

Monash Centre for Health Research and Implementation

Locked Bag 29

Clayton

Victoria

Dr Genevieve Steiner

NICM Health Research Institute

Western Sydney University Campbelltown Campus

Building 22

Campbelltown

NSW

Prof Nigel Stepto

Victoria University

Melbourne

Victoria

Ms Adele Cave

NICM Health Research Institute

Western Sydney University Campbelltown Campus

Building 22

Campbelltown

NSW

Ms Jie Hao

NICM Health Research Institute

Western Sydney University Campbelltown Campus

Building 22

Campbelltown

NSW

**ROLES OF INVESTIGATORS**

CI Ee is the lead investigator and oversees the day to day conduct of the trial including overseeing research staff, checking CRFs for completion, training and overseeing the study acupuncturists, overseeing recruitment, and data collection and analysis. CI Ee screens all women for diagnosis of PCOS, including making the diagnosis if required; provides information about acupuncture in order for women to provide informed consent; and reviews participants medically should there be an adverse event.

CIs Smith, Costello, Moran, Teede, Stepto, and Steiner contributed to the design of the study. In particular, CI Smith provides input into acupuncture aspects of the study, CIs Teede and Costello provide additional medical input into the study (such as decisions about eligibility), and CIs Stepto and Moran provide input into lifestyle aspects of the study (such as advising on physical activity data collection and analysis), CI Steiner oversees heart rate variability data collection and analysis.

CI Cave is a research assistant on the study and conducts the clinic visits including: Obtaining informed consent, obtaining anthropometric measurements, administering surveys, conducting heart rate variability assessments.

Jie Hao provides additional research assistance, assisting with anthropometric measurements, data cleaning and analysis.

**DECLARATION OF INTERESTS**

CE declares that she is a practising acupuncturist. As a medical research institute, NICM Health Research Institute receives research grants and donations from foundations, universities, government agencies, and industry. Sponsors and donors provide untied and tied funding for work to advance the vision and mission of the Institute.

**STUDY CENTRE**

NICM Health Research Institute

Western Sydney University Westmead and Campbelltown campuses

NSW

Phone: 02 46203290

**SITES**

Hills Family Acupuncture @ Hills Allied Health

Suite 208a Q Centre,

10 Norbrik Drive,

Bella Vista 2153

Completely Aligned

Shop 10, Camden Village Court

180-186 Argyle St

Camden 2570

The Acupuncture Pregnancy Clinic

Level 2, Suite 104,

15 Bowden St Alexandria (Alexandria Specialist Day hospital)

Alexandria 2015

The Acupuncture Pregnancy Clinic

Suite 35, The Daher Centre 163 Hawkesbury Rd

Westmead 2145

The Dao Health

5/345 Kingsway

Caringbah 2229

Lisa Holden

12 Singles Ridge Rd

Winmalee 2777

Study acupuncturists are responsible for: Delivering acupuncture or AEA treatments according to protocol, administering the credibility questionnaire, completing the consultation case report forms, and collecting information about adverse events

**TABLE OF CONTENTS**

*1* Introduction 8

1.1 Background 8

1.2 Rationale for Study 9

2 Study Objectives 9

2.1 Objectives 9

2.1.1 Primary Objective 9

2.1.2 Secondary Objectives 9

2.2 Endpoints 9

2.2.1 Primary Endpoint 9

2.2.2 Secondary Endpoints 10

3 Study Design 10

4 Study Population 10

4.1 Number of Participants 10

4.1.1 Inclusion Criteria 10

4.1.2 Exclusion Criteria 10

5 Participant Selection And Enrolment 11

5.1 Identifying Participants 11

5.1.1 Recruitment sources 11

5.1.2 Procedure for identifying participants 11

5.2 Consenting Participants 13

5.3 Screening for Eligibility 13

5.4 Ineligible and Non-Recruited Participants 13

5.5 Randomisation Procedures 13

5.5.1 Randomisation 13

5.5.2 Withdrawal procedures 14

6 intervention and Control 14

6.1 Therapeutic Intervention: Body acupuncture 14

6.1.1 Acupuncture rationale 14

6.1.2 Details of needling 15

6.1.3 Treatment regimen 15

6.1.4 Other components of treatment 16

6.1.5 Practitioner background 16

6.2 Therapeutic intervention 2: Auricular Electro-acupuncture stimulation (AEA) 17

6.2.1 Acupuncture rationale 17

6.2.2 Details of needling 17

6.2.3 Treatment regimen 17

6.2.4 Other components of treatment 18

6.2.5 Practitioner background 18

6.3 Control intervention 18

6.4 Co-interventions 19

6.4.1 Lifestyle intervention 19

7 Study Assessments 19

7.1 Study Assessments 19

7.1.1 Study Procedures 19

8 Data Collection 20

8.1 Anthropometric measures and blood pressure 21

8.2 Measures of insulin resistance 21

8.3 Heart Rate Variability 21

8.4 Other outcomes collected during clinic visits 22

8.5 Credibility, expectancy and perceived treatment allocation 22

8.6 Other outcomes 22

9 Statistics and Data Analysis 22

9.1 Sample Size Calculation 22

9.2 Proposed Analyses 22

10 Adverse Events 22

10.1 Definitions 23

10.2 Detecting AEs and SAEs 24

10.3 Managing potential risks 24

10.4 Managing adverse outcomes 25

10.5 Recording AEs AND SAEs 26

10.6 Evaluation OF AEs AND SAEs 26

10.6.1 Assessment of Seriousness 26

10.6.2 Assessment of Causality 26

10.6.3 Assessment of Severity 27

10.6.4 Assessment of Expectedness 27

10.7 Reporting of SAEs/SARs/SUSARs 28

10.8 Follow up Procedures 28

11 Good Clinical Practice Module 28

11.1 Ethical Conduct of the Study 28

11.2 Investigator Responsibilities 29

11.3 Informed Consent 29

11.4 Emergency Contact with Investigators 29

11.5 Notification of Primary Care Physician 29

11.6 Investigator Indemnification 30

11.7 Study Site Staff 30

11.8 Data Recording 30

11.8.1 Confidentiality 30

11.8.2 Data Protection 30

12 Study Administrative Procedures 32

12.1 Protocol Amendments 32

12.2 Protocol Violations and Deviations 32

12.3 Study Record Retention 32

12.4 End of Study 32

13 Reporting, Publications and Notification of Results 33

13.1 Authorship Policy 33

13.2 Publication 33

14 Appendix 1 - Schedule of Observations and Procedures 34

15 References 36

**Summary**

*Professional Summary*

This feasibility study on acupuncture for weight loss in Polycystic Ovary Syndrome (PCOS) is a collaboration between the National Institute of Complementary Medicine/Western Sydney University, the PCOS Centre for Research Excellence, Jean Hailes for Women’s Health, GetHealthyNSW and IVF-Australia and aims to assess feasibility and acquire data to inform a fully-powered trial for submission to nationally competitive funding agencies. This study is also a partnership with Neural Ear Stimulation International (NESI Corp).

*Lay Summary*

Polycystic Ovary Syndrome (PCOS) is a common women’s health hormonal problem that can result in irregular periods, infertility, weight gain, acne and excess body hair, and anxiety and depressive symptoms. Weight loss can improve these symptoms, however many women find this difficult to achieve. Acupuncture is a Chinese medical treatment that involves insertion of very fine metal needles into specific areas of the body, and has been shown to help obese and overweight adults lose weight, however few studies have been conducted in women with PCOS. A variant of acupuncture, called auricular electro-acupuncture (electrical stimulation to points on the ear) may have beneficial effects on "fight or flight" syndrome which is associated with PCOS. This study aims to collect preliminary information on whether it is possible and acceptable to perform clinical research on acupuncture and/or auricular electro-acupuncture for weight loss in PCOS women. We are partnering with IVF-Australia, a leading fertility organisation in Australia, to recruit women for this study. We aim to analyse 30 women in this clinical trial. Women will be randomly allocated to receive one of three types of treatments: acupuncture as well as a telephone-based lifestyle coaching; AEA and telephone-based lifestyle coaching; or telephone-based lifestyle coaching alone. We will measure women’s weight before and after treatment and investigate the possible ways that acupuncture and AEA may benefit weight in PCOS, including measuring heart rate variability and insulin levels. The findings from this study will help us design a larger trial that will provide more definitive answers as to whether acupuncture or AEA is an effective treatment for weight loss. If acupuncture helps women with PCOS lose weight, this means that these women will have improved chances of having a baby, more regular periods, and a lower risk of developing diabetes and heart disease in the future.

**Study Synopsis**

| **Title:** | **Acupuncture or auricular electro-acupuncture as adjuncts to lifestyle interventions for weight management in PCOS: a randomised controlled feasibility study** |
| --- | --- |
| **Sponsor:** | **NHMRC CRE in PCOS (Project Grant)** |
| **Protocol Number:** | **V12** |
| Development Phase: | Phase II |
| Indication: |  |
| **Objectives of the Study:**  Primary Objective: | Determine feasibility and acceptability of study procedures |
| Secondary Objective(s): | Determine an effect size for acupuncture and lifestyle vs AEA and lifesyle vs lifestyle alone and examine for a trend between groups for change in body weight, BMI, Heart Rate Variability and insulin sensitivity |
| **Study Drugs/Therapeutic Intervention:** | Acupuncture and telephone-based lifestyle intervention compared to AEA and telephone-based lifestyle intervention compared to telephone-based lifestyle intervention alone |
| **Study Design**: | Randomised controlled feasibility study |
| **Study Endpoints** (primary and secondary) | Primary: Recruitment, retention, adherence, acceptability , credibility and safety over the recruitment and intervention periods.  Secondary: Difference between groups for anthropometric measures, blood pressure, health-related quality of life, menstrual cycles, psychological measures, self-efficacy and locus of control, heart rate variability, insulin, glucose, Sex Hormone Binding Globulin, total and free testosterone and free androgen index |
| **Sample size** (by treatment group):  Sample Size Determination: (If applicable) | We aim to analyse 30 women (10 in each group) at the end of treatment |
| **Total Number of Study Centres** | 1 |
| **Study Duration:** | 12 weeks |
| **Inclusion Criteria:** | - women aged 18-45 years - physician diagnosis of PCOS according to the 2003 Rotterdam Criteria in previous 5 years[14]; - BMI≥25 kg/m^2^; no other endocrine disorders; - not pregnant/no pregnancies in preceding 6 weeks and more than 6 weeks postpartum; - not on the following medications in the 3 months preceding enrolment: metformin or other medications affecting insulin and glucose metabolism, hormonal treatments for PCOS/ART including gonadotrophins and the Oral Contraceptive Pill, pharmaceutical or complementary therapies for weight loss |
| **Exclusion Criteria:** | - Planning to conceive within the next 3 months - Needle acupuncture in the previous 3 weeks - Unable to provide informed consent. - BMI >40 - anticoagulant use - pacemaker use - immunocompromise - valvular heart disease - Currently breastfeeding or have ceased breastfeeding within the past 6 weeks ( a waiting period may apply) |
| **Dosage Regimen/Duration of Treatment:** | 12 treatments of acupuncture over 12 weeks, twice a week for 4 weeks then fortnightly after |
| Investigational Product/Therapy 1: | Acupuncture and telephone-based lifestyle interventions |
| Investigational Product/Therapy 2: | Auricular Electro-acupuncture (AEA) stimulation and telephone-based lifestyle interventions |
| Control Group: | Telephone-based health coaching |
| **Study Procedures:** | The majority of outcomes will be completed at the pre and post intervention visit. Participants will wear an accelerometer at -1 and 11 weeks for 5-7 days and will complete a menstrual calendar. |
| **Statistical Analyses:** (Brief Description) | Descriptive statistics for primary endpoint. |

# Introduction

## Background

This feasibility study on acupuncture for weight loss in Polycystic Ovary Syndrome (PCOS) is a collaboration between NICM/Western Sydney University, the PCOS Centre for Research Excellence, Jean Hailes for Women’s Health, GetHealthyNSW and IVF-Australia and aims to assess feasibility and acquire data to inform a fully-powered trial for submission to nationally competitive funding agencies. A Partnership with NESI Corp is allowing for the addition of a third comparative arm, using auricular electro-acupuncture.

***Polycystic Ovary Syndrome and obesity/overweight:***

PCOS is a common women’s health condition with significant reproductive, metabolic and psychological manifestations such as infertility and Type 2 Diabetes (T2D)[1]. Women with PCOS are more likely to be obese/overweight than age-matched controls[2], and excess weight worsens the features of PCOS[3]. Weight loss is a key goal in PCOS, and lifestyle management (diet, exercise, and/or behavioural interventions) targeted at weight loss are first-line recommendations in overweight/obese women[3] as even modest weight loss improves reproductive and metabolic outomes. Current evidence-based guidelines on PCOS recommend that obese women embark on 3-6 months of lifestyle management for weight loss prior to considering Assisted Reproductive Technology (ART). However, adherence is generally low[3], and achieving adequate weight loss remains a significant challenge [2].

**The potential role of acupuncture**

In non-PCOS populations, acupuncture is more efficacious than sham for weight loss, with mean differences in body weight and Body Mass Index(BMI) of 1.58kg[4] to 4.4kg[5], and 0.6 kg/m^2^[4]to 2.79 kg/m^2^[6] respectively (3.5-5% decrease from baseline)[4-7]. These effects are mediated through alterations in obesity-related peptides, appetite regulation, insulin levels, free fatty acids and inflammatory markers[8] and are sustained after end-of-treatment[5]. RCTs conducted in China indicate that acupuncture + metformin is superior to metformin alone in women with PCOS for reducing BMI[9]. The overall effect size is unclear, although one study reported a mean difference of 0.97kg/m^2^ (95% CI 1.51, 0.43) for acupuncture + metformin compared to metformin alone[10].

**Could acupuncture be an effective adjunctive weight loss treatment via modulation of sympathetic tone?**

Although the exact etiology of PCOS is unknown, *insulin resistance* (IR) is a key factor and contributes to obesity [13]. *Increased sympathetic tone* is an associated factor of IR [14] and has been identified as a potential therapeutic target in PCOS[15]. In rats with steroid-induced PCOS, electro-acupuncture reduces ovarian sympathetic hyper-innervation, ameliorates IR, and improves oestrous cycling[16]. Electro-acupuncture reduced Muscle Sympathetic Nerve Activity in PCOS women in one trial[14]. Our qualitative work indicates high levels of acceptance of acupuncture as a possible adjunct to lifestyle interventions for weight loss, and subsequently we have secured external funding and Ethics approval for a feasibility study comparing acupuncture and lifestyle interventions (Phase 2b of our research program; see Figure 1; n=20)

**Auricular Electro Acupuncture stimulation: a novel approach to delivering protracted acupuncture?**

A variant of acupuncture is Auricular Electro-Acupuncture (AEA). Our partner has shown that AEA (delivered by the Neurova device) is effective for hot flushes from androgen-deprivation therapy, a condition underpinned by sympathetic hyperactivity[1]. This method involves insertion of indwelling needles into the concha of the ear and delivery of intermittent electrical impulses over 96 hours by a small battery-powered device that is worn just below the ear. This significantly increases the "dose" of acupuncture that can be delivered ("protracted acupuncture").

Evidence suggests that AEA stimulates the auricular branch of the vagus nerve, *increases parasympathetic tone*[18], suppresses the appetite[17] and produces *sympathetic deactivation* [19]. Through this reasoning we expect to improve autonomic function that contributes to improved IR and resultant weight loss.

**Heart Rate Variability: a surrogate marker of autonomic tone**

Sympathetic tone can be measured directly using microneurography; however, this procedure limited by its invasive nature. Heart rate variability (HRV) provides non-invasive measures of both parasympathetic (high frequency/HF component) and sympathetic tone (low frequency/LF component) [16, 18]. Several sham-controlled RCTs on acupuncture for a variety of clinical conditions suggest that it is more efficacious than sham in reducing sympathetic tone as measured by HRV[18-20]. To date, the effect of acupuncture on HRV in women with PCOS has not been evaluated, although electro-acupuncture improved HRV and restored oestrous cycling compared to sham handling in a recent rat study[21].

Our partner's previous research showed that AEA resulted in improvement in HRV LF/HF ratios over the course of the protocol intervention, implying improved sympathovagal balance[1], however this was measured using ambulatory HRV monitoring, which has not been validated against the gold-standard of electrocardiogram (ECG) recordings.

## Rationale for Study

Acupuncture may represent a low-risk[11] non-pharmacological adjunct to lifestyle interventions, but to date no sham-controlled trials have been conducted on acupuncture for weight loss in PCOS. Many randomised controlled trials (RCTs) fail due to inability to recruit to target[12]. To this end, the UK Medical Research Council recommends assessment of feasibility prior to full evaluation of complex interventions[13]. This can ensure that money spent on expensive trials is not wasted due to recruitment and retention failures.

PCOS is the leading cause of anovulatory infertility, and obesity increases the prevalence of infertility and pregnancy complications, as well as metabolic complications such as T2D and cardiovascular disease. Management of these complications is estimated at $800 million annually in Australia[1].

# Study Objectives

## Objectives

### Primary Objective

(1) Assess recruitment, retention and adherence rates and acceptability of trial procedures

### Secondary Objectives

2) Determine an effect size for acupuncture and lifestyle vs lifestyle alone and examine for a trend between groups for change in body weight and BMI.

3) Explore the impact of body electro-acupuncture and auricular electro-acupuncture on sympathetic tone and insulin resistance

## Endpoints

### Primary Endpoint

Recruitment, retention and adherence rates; credibility; acceptability; and safety over the recruitment and intervention periods.

### Secondary Endpoints

Difference between groups for anthropometric measures, health-related quality of life, menstrual cycles, psychological measures, self-efficacy and locus of control; measures of insulin resistance; and heart rate variability; free and total testosterone and free androgen index.

# Study Design

This is a prospective open-label parallel randomised controlled feasibility study taking place over a 12 week intervention period.

# Study Population

## Number of Participants

We aim to analyse 30 participants by the end of treatment. Allowing for a 25% dropout rate which has been reported in similar studies, we will randomise 39 women (13 to real acupuncture, 13 to AEA and 13 to lifestyle alone).

### Inclusion Criteria

- women aged 18-45years;
- physician diagnosis of PCOS according to the 2003 Rotterdam Criteria in previous 5 years [14];
- BMI≥25 kg/^2^; no other endocrine disorders;
- not pregnant/no pregnancies in preceding 6 weeks and more than 6 weeks postpartum;
- not on the following medications in the 3 months preceding enrolment: metformin or other medications affecting insulin and glucose metabolism, hormonal treatments for PCOS/ART including gonadotrophins and the Oral Contraceptive Pill, pharmaceutical or complementary (nutritional/herbal) treatments for weight loss

### Exclusion Criteria

- Planning to conceive within the next 3 months
- Needle acupuncture in the previous 3 weeks
- Unable to provide informed consent.
- BMI >40
- anticoagulant use
- pacemaker use
- immunocompromise
- valvular heart disease
- Currently breastfeeding, or breastfeeding within the last 6 weeks

# Participant Selection And Enrolment

## Identifying Participants

### Recruitment sources

Women living in Sydney will be recruited via

- IVF-Australia fertility clinics and specialty PCOS clinics (brochures and fliers displayed at clinics, and recruitment by clinicians who wish to be involved);
- PCOS Association of Australia,
- Jean Hailes for Women’s Health and other women’s health or health or community online sources
- Fliers at strategic places in the community such as fitness centres
- social media
- Gethealthy NSW
- Study webpage hosted on the National Institute of Complementary Medicine’s website with a description of the trial, the Participant Information and Consent Form, Ethics approval details, contact details for the Research assistant and for CI Ee, and a link to an online survey where participants can self-screen for eligibility
- Western Sydney University staff and student portals

### Procedure for identifying participants

A proxy website will be created with a memorable URL that will redirect potential subjects to the official study webpage. A Facebook page will be created that will post study updates and contain a link to the study webpage.

Women will be either be invited to the study by a clinician working at IVF-Australia clinics, or self-refer after viewing details on brochures and fliers displayed in clinics, and online advertisements via Jean Hailes, PCOS Association, social media and other online sources such as university newsletters. Women can complete an online survey to self-screen, or be screened over the telephone by the research assistant.

Clinicians who agree to be involved in recruitment at IVF-A will be provided with brief information about the study and a brochure inviting the patient to take part. Clinicians will identify potentially eligible women either at the time of consultation, or through an audit of clinic patients. Patients identified by audit will be mailed a letter from the clinician with brief details about the study, and a brochure and the Participant Information and Consent Form, and will be encouraged to call, email or self-screen using the online survey to check eligibility.

GetHealthyNSW telephone operators will be provided brief details about the study, eligibility criteria, and will invite potentially eligible callers to the study by providing them with brief information and obtaining consent for the research assistant to contact the participant by phone or email. They will also be provided with the online survey link in order to self-screen if they wish.

Brochures and fliers will also be displayed at Western Sydney University campus sites, GP clinics and other community sites such as fitness centres and community health centres.

Withdrawal criteria: Women can withdraw at any time and their data will be removed from the study upon their request.

## Consenting Participants

At the time of confirming eligibility, women are provided with Participant Information and Consent Form and are also provided with verbal information about the trial from either CI Ee or the Research Assistant. Participants are given sufficient time to read the PICF and ask questions. It is anticipated that in the most part participants will be provided the PICF by email or mail, and verbal information over the telephone. Following which an appointment is made for the baseline clinic visit OR the participant is given time to make a decision and the Research Assistant will contact her within a week to enquire of the decision. Once the participant decides to enrol in the trial, verbal consent is obtained in order to start collecting data for the run-in period which includes having a blood test for an OGTT, and wearing an accelerometer for 5-7 days prior to the baseline visit.

At the baseline/pre intervention clinic visit, participants are given another opportunity to ask questions prior to providing written consent to participate in the trial. At that point, the participant is enrolled into the trial and is assigned a study ID number and is randomised.

Written consent will be obtained either by the Research Assistant or CI Ee. If CI Ee is not able to be present at the time of written consent, she will telephone the participant beforehand to answer any questions that pertain to acupuncture treatment and risks.

## Screening for Eligibility

Participants have to meet the eligibility criteria described in 4.1.1 and 4.1.2. This is performed using the Screening Survey which can be administered over the telephone by the Research Assistant, or self-reported using a paper or electronic survey. Potential subjects may self-screen using the online survey. If they are eligible, they are asked to provide contact details (name, and two telephone numbers, and an email address). The research assistant will then contact the participant initially by email (providing the participant with details of the next steps, the PICF, and contact details). The research assistant will also call the participant to arrange a baseline/pre intervention clinic visit.

Enrolment into the trial requires diagnosis of PCOS by a physician in the previous five years. Women are asked to confirm if they have been diagnosed by a physician in the last five years (GP, Endocrinologist, Gynaecologist) and if they meet the Rotterdam criteria. Dr Ee will make contact with all potentially eligible participants following completion of the screening survey, and confirm the diagnosis, which may involve viewing de-identified ultrasound or pathology reports. Women will be asked to de-identify their own reports by removing all identifiers on their report such as name, date of birth, and address using a marker pen. They will then fax or email the de-identified reports to Dr Ee's university email address or office fax. Alternatively, they can request for pathology providers or radiology providers to securely transmit reports to Dr Ee's private GP clinic. This process mimics usual clinical practice and is convenient and secure.

It is unlikely that women will need to provide previous reports as the majority of them are anticipated to be diagnosed according to clinical criteria (menstrual irregularity, signs of hyperandrogenism such as hirsutism or male pattern balding). If women do not have reports within the last five years and do not have current signs or symptoms of PCOS, they will be asked to see their GP to have the diagnosis confirmed by pathology/ultrasonography, or refuted. This is relevant as some women may no longer have PCOS (for example, if they lose weight) and therefore will not require treatment.

The diagnosis is made or confirmed by Dr Ee if women are currently still having symptoms and signs of PCOS (i.e. oligomenorrhoea and/or clinical hyperandrogenism), and were symptomatic at the time of having abnormal pathology and radiology reports (if relevant).

The medical records that women may need to obtain for Dr Ee's perusal include:

- testosterone, free testosterone, Sex hormone binding globulin, free androgen index levels
- pelvic ultrasound reports
- Thyroid function tests

Rotterdam Criteria:

Two out of three of:

a. Menstrual cycles greater than 35 days apart OR shorter than 21 days apart^1^

b. Clinical (hirsutism, male pattern balding) or biochemical hyperandrogenism^2^ (testosterone and/or free androgen index)

c. Polycystic ovaries on ultrasonography when >18yo^3^ ( ten or more small antral follicles in both ovaries)

^1^Thyroid disorders should be excluded

^2^ If androgen levels are markedly elevated (testosterone levels >20% over upper limit) or there was rapid or sudden onset of hyperandrogenism, congenital adrenal hyperplasia or rarer causes of hyperandrogenism should be excluded by performing a serum 17-hydroxyprogesterone level in the follicular phase and also by endocrinology review

^3^If ultrasonography was conducted when the woman was an adolescent, results are to be interpreted with caution. Vaginal ultrasounds are desirable, but abdominal US can be considered for use in diagnosis.

If participants are screened by the research assistant, this is done over the telephone and if eligible the participant is invited to attend for a baseline clinic visit.

Participants are also required to have an Oral Glucose Tolerance Test (OGTT )done as part of the outcomes of the study. The baseline OGTT will be done prior to enrolling the participant, as diagnosis with Type 2 diabetes or impaired glucose tolerance (both of which can be diagnosed from the OGTT) is an exclusion criterion. CI Ee will contact the participant to inform them of their results and eligibility to join the trial. If the participant is diagnosed with diabetes or impaired glucose tolerance, they are referred to their GP for further management, and are not eligible to enrol for the trial.

All women will be offered the opportunity to have their diagnosis of PCOS re-confirmed through blood tests done at baseline. This is *only* offered to women who have had an initial diagnosis of PCOS made by a doctor at any stage. Women are offered the opportunity to have the following blood tests to confirm the diagnosis: TSH and/or Prolactin, Free Androgen Index, Free and Total Testosterone. The latter two tests are already performed at baseline, but they can be used as a screening test if required.

## Ineligible and Non-Recruited Participants

Participants who are not eligible or decline consent will be thanked for their involvement and interest in the study, and will be asked for consent to be contacted again should eligibility criteria change. Participants can opt to complete a 3 month washout if applicable prior to enrolling.

## Randomisation Procedures

### Randomisation

Women will be randomised in a 1:1 ratio to receive either acupuncture + lifestyle, AEA + lifestyle, or lifestyle alone. Randomisation will be performed in permuted blocks of six.

#### Sequence generation

The randomisation sequence will be created using a computer program by a researcher external to the research team. This researcher will hold the randomisation sequence and will create a series of 39 consecutively numbered sealed opaque envelopes that will contain the ID number and allocation.

#### Allocation concealment

The research assistant will allocate participants by selecting the next consecutively number sealed opaque envelope that contains the allocation.

#### Implementation

Once the participant has had eligibility confirmed and has provided written informed consent, she is enrolled and given the next consecutive randomisation ID number, and the research assistant will open the numbered envelope to reveal the allocation. Before opening the envelope the research assistant writes the participant's name on the envelope, the date, and signs the envelope as a record of randomisation. The research assistant will inform the treating acupuncturist of the new enrolment and book the participant’s first appointment.

#### Blinding

Investigators (outcome assessors and investigators involved in statistical analysis) are blinded to treatment allocation but treating acupuncturists and participants are not.

### Withdrawal procedures

Participants are free to withdraw at any time during the study. If they wish to withdraw they can notify either their acupuncturist or the research assistant or CI Ee. The acupuncturist is to then report the withdrawal to CI Ee. The research assistant or CI Ee will call the participant to thank them for their involvement and enquire about the reason for wishing to withdraw although if the participant does not wish to provide a reason or have any further contact with the research team, the research team will respect this wish. If the participant is open to this, the RA or CI Ee will discuss methods that will assist the participant to continue on with the trial (for example, changing acupuncturist location if this is more convenient). If the participant does not wish to discuss this, it will not be pursued. The participant will be asked if she would be willing to attend for the post-intervention clinic visit or complete the post-intervention surveys. Participants will not be replaced once they are withdrawn.

Data for the withdrawn participant will be used in the analysis unless the participant requests for it to be also withdrawn.

# intervention and Control

## Therapeutic Intervention: Body acupuncture

### Acupuncture rationale

#### Style of acupuncture

The intervention in this study is Chinese medicine needle acupuncture delivered in a semi-pragmatic setting (as per usual clinical practice but treating acupuncturists must include acupuncture points from a list provided).

#### Reasoning for treatment provided

Acupuncturists are to perform Chinese medicine diagnosis based on history and examination as per usual clinical practice. The list of points that acupuncturists must select from are based on acupuncture points used in other clinical trials on acupuncture for weight loss in PCOS and also on a textbook and literature review.

#### Extent to which treatment will be varied

In choosing the acupuncture prescription, treating acupuncturists are given the following instructions:

- Treatment of obesity/overweight is the primary objective of the acupuncture treatment
- At least six acupuncture points must be chosen from a list of 17 core acupuncture points that will be provided to the practitioner. These points are chosen from the traditional points that are recommended for treatment of syndromes that are related to obesity and PCOS, and from the points used in previous acupuncture for obesity and PCOS trials.
- Points on the chest or back must be avoided to avoid risk of pneumothorax.
- *De qi* must be obtained for each point using thrusting, twirling and rotating, until the participant reports numbness, heaviness, pressure, soreness or tingling.
- Needles are manipulated after ten minutes (thrusting, twirling and rotating until De qi is reported)
- Needles are withdrawn after 30 minutes

### Details of needling

#### Number of needle insertions per subject per session

A minimum of ten acupuncture needles will be inserted per session.

#### Names of points to be used

The core acupuncture points to be used are ST36, SP6, ST25, ST40, CV12, CV6, LI11, SP9, CV9, CV3, CV4, LI4, KI13, KI7, ST28, LR3, GB34. These points are located on the upper or lower limbs or abdomen. Acupuncture points will be generally needled bilaterally, unless they are midline points, or there are other reasons for unilateral needling for example pain or bruising on one side.

#### Depth of insertion and response sought

Needles will be inserted until *de qi* is obtained. *De qi* is defined as ***numbness, heaviness, pressure, soreness or tingling.***

#### Needle stimulation

A combination of manual and electrical stimulation (electro-acupuncture) will be used. *De qi* must be obtained for each point using thrusting, twirling and rotating, until the participant reports numbness, heaviness, pressure, soreness or tingling. Electro-acupuncture will be applied to ST28 and SP6 bilaterally (4 points, on the lower abdomen and lower leg). Electro-acupuncture is delivered using TGA-approved electro-acupuncture devices that deliver low-frequency electrical pulses to the attached needles (2Hz, 0.3ms, continuous stimulation) and intensity is to be gently increased until a small muscle contraction is seen or felt, but without any discomfort or pain. Acupuncturists are to note down the intensity of the stimulation at each session.

#### Needle retention time

Needles are retained for 30 minutes.

#### Needle type

Needles are stainless steel, sterile and disposable. Acupuncture needles are standard , 32G thickness and 30mm length.

#### Quality control

Each acupuncture practitioner is to take a digital photo of the acupuncture points inserted in order for acupoint location to be verified. This is done once per practitioner, and within the first few treatments provided for the trial. The de-identified photo is to be sent to CI Ee by text message or email and identified only by ID number and date of treatment, and will be stored on the University’s secure cloud server. No identifying features will be included (such as facial features). Privacy must be maintained by using sheets or blankets to cover any sensitive areas such as the pubic region. Consent from each participant is sought at the time of enrolment.

### Treatment regimen

#### Treatment frequency and duration

Women allocated to the acupuncture group will receive a total of 11 treatments of acupuncture over 12 weeks (twice a week for the first two weeks, weekly for four weeks, then fortnightly for 3 treatments). This tapering schedule is consistent with usual clinical practice. Needles are retained for 30 minutes.

#### Dealing with missed sessions

At the time of enrolment, participants are to be made aware of the nature of their commitment over the twelve week treatment period and encouraged to notify the research office, CI Ee or the treating practitioner as soon as a break in treatment is foreseen. If treatments are missed, all attempts will be made to complete ten treatments within the eight weeks, providing there is at least a 2 day interval between treatments A break in the intervention schedule may be unavoidable, due to illness or holiday. If the acupuncturist becomes aware that a break of more than two weeks is likely, they will be instructed to liaise with CI Ee for advice on how to deal with this. CI Ee or the research assistant will liaise with the participant to determine the cause of missed sessions and will attempt as much as possible to implement appropriate measures to minimise further breaks in between treatments.

As analysis will be by intention-to-treat (that is, all data will be analysed even if a participant has dropped out from the trial), a minimum number of treatment sessions to meet the protocol has not been specified.

### Other components of treatment

Treating acupuncturists may provide diet and lifestyle advice according to Chinese medicine principles. This advice usually consists of advice to avoid ingesting foods that are considered too “hot” or “cold” for the patient’s constitution. However, no additional Chinese medicine treatments will be provided including Chinese herbal medicine, or additional physical or stimulation treatments such as moxibustion or cupping.

Participants will attend for treatment at private clinics of the treating acupuncturists. Treating acupuncturists are advised that they may proceed according to usual clinical practice with the following limitations:

- The acupuncture protocol must be targeted at weight loss as the primary outcome
- The acupuncture protocol must adhere to the standardization applied to acupuncture points, manipulation, stimulation, retention time, frequency as described above
- Only body acupuncture, diet and lifestyle advice (according to Chinese medicine) may be provided.

Practitioners are free to practise as they usually practise. They will be trained to ask about adverse events, and to treat patients warmly and courteously. All efforts will be made to ensure the participants have a comfortable acupuncture experience.

Practitioners will record details of each treatment on an individual Case Report Form which will be kept in a locked cupboard in the clinic and returned to the research team at the end of the study.

### Practitioner background

A minimum of six practitioners have been chosen to administer the treatments. They are have a Bachelor degree in Chinese Medicine, have five or more years of clinical experience and are registered with the Chinese Medicine Registration Board.

Practitioners will receive training from CI Ee, an experienced acupuncture researcher. Training will take place 4-6 weeks prior to anticipated start of recruitment. They will receive a detailed Practitioner Training Manual. Training will be “hands on”. Refresher training will be provided if there has been a break of two months or more in between administering the trial interventions to participants.

Participants are free to choose which practitioner they wish to see according to convenience (location, availability etc) however they are to be aware that there may be a “waitlist” for particular practitioners. Practitioners may also notify the research assistant or CI Ee when they are unable to take on new participants temporarily due to other commitments.

## Therapeutic intervention 2: Auricular Electro-acupuncture stimulation (AEA)

### Acupuncture rationale

#### Style of acupuncture

The intervention in this study is auricular electro-acupuncture using a fixed protocol that is informed by both Chinese medical and medical acupuncture principles.

#### Reasoning for treatment provided

No Chinese medicine diagnosis is required. The acupuncture points are chosen based on a literature review and expert consensus on the traditional and physiological indications of the points.

#### Extent to which treatment will be varied

There is to be no variation in treatment.

### Details of needling

#### Number of needle insertions per subject per session

Each subject will receive needling at three acupuncture points on the concha of one ear only. The ears are to be alternated between visits unless there is any objection from the participant.

#### Names of points to be used

The core acupuncture points on the concha of the ear to be used are the Stomach, Appetite Control, and Thalamus points.

#### Depth of insertion and response sought

No *de qi* sensation is to be sought. Needles are to be inserted superficially and not into the cartilage of the ear.

#### Needle stimulation

The Neurova device will be attached after insertion of the needles to the ear, according to the manufacturer's instructions (See Appendix 15).

#### Needle retention time

Needles are retained for up to 96 hours. The participant is to remove the Neurova device by gently pulling out the needles and disconnecting the wire. The needles are then placed into a provided sharps container. Gentle pressure is applied with a cotton ball if there is any bleeding. Participants can remove the device before 96 hours if they experience significant discomfort. Participants bring the sharps container back to the post-intervention visit for adequate biological hazard waste disposal through NICM laboratories.

#### Needle type

Needles are surgical grade titanium, sterile and disposable and approximately 6mm in length.

### Treatment regimen

#### Treatment frequency and duration

Women allocated to the AEA group will receive a total of 6 treatments of acupuncture over 12 weeks (twice a week for the first two weeks, then fortnightly for four treatments).

#### Dealing with missed sessions

At the time of enrolment, participants are to be made aware of the nature of their commitment over the twelve week treatment period and encouraged to notify the research office, CI Ee or the treating practitioner as soon as a break in treatment is foreseen. If treatments are missed, all attempts will be made to complete ten treatments within the eight weeks, providing there is at least a 2 day interval between treatments A break in the intervention schedule may be unavoidable, due to illness or holiday. If the acupuncturist becomes aware that a break of more than two weeks is likely, they will be instructed to liaise with CI Ee for advice on how to deal with this. CI Ee or the research assistant will liaise with the participant to determine the cause of missed sessions and will attempt as much as possible to implement appropriate measures to minimise further breaks in between treatments.

As analysis will be by intention-to-treat (that is, all data will be analysed even if a participant has dropped out from the trial), a minimum number of treatment sessions to meet the protocol has not been specified.

### Other components of treatment

No additional Chinese medicine treatments will be provided including body acupuncture, Chinese herbal medicine, or additional physical or stimulation treatments such as moxibustion or cupping.

Participants will attend for treatment at private clinics of the treating acupuncturists**.** Practitioners are trained to ask about adverse events, and to treat patients warmly and courteously. All efforts will be made to ensure the participants have a comfortable acupuncture experience.

Practitioners will record details of each treatment on an individual Case Report Form which will be kept in a locked cupboard in the clinic and returned to the research team at the end of the study.

### Quality assurance

As part of monitoring of treatment fidelity and quality assurance, practitioners are requested to take a digital photo of the first insertion of the Neurova that they perform, to be verified by the Chief Investigator and by NESI Corp (the manufacturers). This photo will not contain any identifying features such as facial features. The photo will be labelled with the participant ID number and date of treatment only, and emailed to CI Ee, who will then store the photograph on a secure University cloud-based server.

### Practitioner background

A minimum of six practitioners have been chosen to administer the treatments. They are have a Bachelor degree in Chinese Medicine, have five or more years of clinical experience and are registered with the Chinese Medicine Registration Board.

Practitioners will receive training from CI Ee, an experienced acupuncture researcher. Training will take place 4-6 weeks prior to anticipated start of recruitment. They will receive a detailed Practitioner Training Manual. Training will be “hands on”. Refresher training will be provided if there has been a break of two months or more in between administering the trial interventions to participants.

Participants are free to choose which practitioner they wish to see according to convenience (location, availability etc) however they are to be aware that there may be a “waitlist” for particular practitioners. Practitioners may also notify the research assistant or CI Ee when they are unable to take on new participants temporarily due to other commitments.

## Control intervention

The control intervention is lifestyle intervention only (see 6.4.1below).

## Co-interventions

Although participants will be encouraged to avoid using new co-interventions, co-interventions including complementary therapies will be allowed.

### Lifestyle intervention

All participants will register for the GetHealthy NSW program. All women will receive individually-tailored telephone-based lifestyle coaching for 3 months, consisting of up to 13 telephone calls from an exercise physiologist with nutrition training, exploring healthy eating, physical activity, and weight management. Lifestyle coaching is provided by Remedy Healthcare under the GethealthyNSW[19] program, which has been offered to all NSW residents for free by the NSW government since 2009. Medical referral is not required. Information given to participants is consistent with national guidelines on physical activity and nutrition, which are also recommended by national PCOS guidelines[3].

# Study Assessments

## Study Assessments

### Study Procedures

Participants will attend for two clinic visits, Baseline/Pre-intervention and Post-intervention.

During these visits they will complete the majority of the outcome measures for the trial.

In addition, the following outcomes are also collected outside of the two clinic visits:

- Credibility of the treatment, expectations, and perceived treatment allocation are collected immediately after the first acupuncture treatment
- Reproductive: Menstrual cyclicity from menstrual diaries
- Participants will complete an exit survey about the acceptability of the study procedures
- Participants will wear an accelerometer for 5-7 days in the week before attending for the baseline visit, and in Week 11
- Participants and treating acupuncturists will collect data on adverse events throughout the study

#### Pre-intervention/ Baseline visit

The purpose of the baseline visit is to obtain informed consent, collect baseline and demographic measures, randomise the participant, and organise the acupuncture treatments for the participant.

*Informed consent*

If the research assistant is not a practising acupuncturist, the participant will be telephoned by CI Ee, an acupuncturist and researcher, in order to provide details about the acupuncture treatment and answer any queries about acupuncture. This is essential so that the participant can provide informed consent after understanding the risks and benefits of acupuncture treatment. This telephone call will take place prior to the baseline clinic visit.

During the clinic visit, which will take place at the National Institute of Complementary medicine, Campbelltown Campus, Western Sydney University, the participant will meet with the research assistant to provide informed consent.

*Baseline and demographic/medical measures*

The participant will have baseline outcome measures taken as per the following section on Data Collection. Briefly they include:

- Anthropometric measures and blood pressure
- Surveys on quality of life, anxiety and depression, weight self-efficacy, and internal locus of control.
- Measures of insulin resistance by blood tests collected by Laverty Pathology
- Testosterone, Free testosterone and Free Androgen Index collected by Laverty Pathology
- Clinical Hyperandrogenism as assessed by the Ferriman Gallwey score
- Heart rate using ECG monitoring at Headbox laboratory on the WSU Campbelltown Campus and also using a hand-held ambulatory monitor at the same time

Participants will also provide demographic and medical history details.

*Randomisation*

Participants will be assigned a randomisation ID number.

*Acupuncture treatments*

Participants are provided with information about the acupuncture treatments, will choose a treating acupuncturist for the intervention, and the research assistant will organise the participants’ acupuncture treatments according to the schedule.

#### Credibility, expectancy and perceived treatment allocation

Immediately following the first treatment, participants will complete the Credibility and Expectancy Questionnaire and question on perceived treatment allocation. This can be done online at the acupuncturist’s clinic or via email, or on paper and returned to the research assistant by fax or email.

#### Post-intervention visit

At the post-intervention visit which takes place after the participant has completed all her acupuncture treatments, the participant completes the same outcome measures as at the baselinepre-intervention visit but without the demographic/medical questionnaire.

#### Other outcomes

- Physical activity levels will be quantified using accelerometers which will be worn by participants in the week before attending for the baseline visit, and in Week 11 (minutes per week of light, moderate and vigorous activity; number of steps per day; number of sedentary hours per day).
- Data on compliance with the lifestyle coaching program and change in dietary habits will be provided by Gethealthy NSW at baseline and 3 months.
- Participants will record the dates of their menstrual cycle using a menses calendar.
- Participants and treating acupuncturists will be provided with Adverse Events forms on which to record adverse events.

# Data Collection

Data on recruitment, retention, adherence and acceptability rates will be entered into Redcap clinical trial management software. These are defined as:

1. Recruitment rates (number of enquiries and number of enrolments per month of active recruitment; percentage conversion to enrolment measured as n enrolled/n of enquiries, and n enrolled/n potentially eligible);
2. Retention rate (n completing 12 week intervention and outcome measures/n enrolled)
3. Adherence rates (n completing at least 8 of 11 acupuncture treatments and 4 of 6 Neurova treatments; n of hours of wear of the Neurova^TM^ device/percentage of women who wore the device for the prescribed 96 hr duration; n completing all recommended phone calls with the GHS);
4. Acceptability is measured using an exit survey which includes questions on perceived benefits of body acupuncture, AEA, and health coaching, perceived detriments of being in the trial, likelihood of recommending the study to family or friends measured on a 5 point Likert scale ranging from “I would definitely participate/recommend it” to “I would definitely not participate/recommend it”;

## Anthropometric measures and blood pressure

We will measure weight on a calibrated digital scale, waist and hip circumference at the baseline and follow-up clinic visits. We will measure height by stadiometer at the baseline clinic visit. Blood pressure is measured while sitting quietly and after resting quietly for a few minutes, and with both feet on the ground, using an Omron digital automatic standard blood pressure monitor. A large cuff is used if required. Blood pressure on both arms will be obtained initially and the higher blood pressure of the two will be used. Three measurements will be taken, and the average of the last two measurements is recorded. If there is >10mm/Hg or >6mm/Hg difference in systolic or diastolic blood pressure respectively, the participant is asked to rest quietly for five minutes and blood pressure measurement is repeated ([48](#_ENREF_48)).

Anthropometric measures and blood pressure will be measured according to standard procedures as outlined in http://www.cdc.gov/NCHS/data/nhanes/nhanes_07_08/manual_an.pdf

Data will be entered by the Research assistant into the Redcap database.

## Measures of insulin resistance

Participants will attend for a 2 hr Oral Glucose Tolerance Test and Insulin test prior to attending for the baseline visit and at the 3-month follow-up. Insulin will be calculated using the Area Under the Curve (AUC) method. Participants will attend for these tests at accredited pathology collection centres (Laverty Pathology).

Biomarkers that are related to insulin resistance and PCOS are collected at the same time and include Sex Hormone Binding Globulin, total and free testosterone.

Abnormal results will be followed up by phone and letter to the participant and GP by Dr Carolyn Ee, who is a practising GP. Participants will be asked to attend their GP for follow-up. If participants are diagnosed with diabetes, they are excluded from the trial.

## Heart Rate Variability

Heart Rate Variability will be calculated from electrocardiogram (ECG) recordings during the clinic visits.

ECG recordings take place in the Headbox labs on the Campbelltown campus, Western Sydney University. Women will be asked to avoid caffeine and smoking for at least two hours prior to HRV measurement. The total time for ECG recordings is up to 30 minutes.

A research assistant, supervised by CI Steiner, an experienced psychophysiologist with substantial expertise in measures of ANS physiology, will conduct ECG recordings. ECG electrodes will be placed on the sternum and lower left ribs. Five minutes of seated resting-state 2-lead ECG will be recorded via bipolar Ag/AgCl electrodes with a Compumedics Neuroscan Synamps 2 Digital Signal Processing System. Interbeat intervals (IBIs) are calculated from the ECG trace before a fast-fourier transformation is then applied to convert HRV data from time to frequency domain. HRV frequency bands will be defined as Very Low Frequency (< 0.04 Hz), Low Frequency (LF; 0.04 – 0.15 Hz), and High Frequency (HF; 0.18 – 0.40 Hz). LF/HF ratio will also be calculated as a measure of sympathovagal balance.

## Clinical Hyperandrogenism

The Ferriman-Gallwey scoring system was developed by Ferriman and Gallwey in 1961 and is widely used to evaluate and quantify hirsutism in women. The method was modified and now incorporates nine body regions (excludes legs and forearms) for the assessment of hair growth, rated from 0 (no growth of terminal hair) to 4 (extensive hair growth) in each of the nine locations. The FG score will be assessed by the research assistant and/or by the woman herself (by indicating on a diagram which of the diagrams best represents her current level of hair growth). {Ferriman, 1961 #4348}

## Other outcomes collected during clinic visits

The following data will be collected during clinic visits. Participants may use an online survey or paper surveys. Data on paper surveys will then be manually entered into Redcap by the Research assistant.

- Health related quality of life – the Modified PCOS Questionnaire
- Global quality of life - EQ-5D
- Psychological Symptoms – the DASS 21
- Weight self efficacy – the Weight Efficacy Lifestyle Questionnaire short form
- Internal locus of control – the Internal Health Locus of Control questionnaire
- Demographic questionnaire – see Appendix

## Credibility, expectancy and perceived treatment allocation

Immediately following the first treatment, participants will complete the Credibility and Expectancy Questionnaire and question on perceived treatment allocation. This can be done online at the acupuncturist’s clinic or via email, or on paper and returned to the research assistant by fax or email.

## Other outcomes

Accelerometers will be collected from participants at the follow-up treatment visit and data will be downloaded electronically.

Data on compliance with the lifestyle coaching program will be provided by Gethealthy NSW at baseline and 3 months.

## Participant Reimbursement

Participants will be reimbursed $40 in total for travel costs associated with the study. At the second and final clinic visit, participants will be offered the opportunity to claim a maximum of $40 by completing a standard invoice template which will be submitted to NICM HRI administration to be processed. The payment is made by direct bank deposit to the participants' nominated bank account.

# Statistics and Data Analysis

## Sample Size Calculation

***Sample size*** has been determined to answer the research questions regarding an estimate of the effect size, and recruitment, retention and adherence rates. 39 participants will be recruited; allowing for 20-25% attrition which has been reported in other studies on weight loss in PCOS, we aim to analyse 30 participants at end of treatment.

## Proposed Analyses

Recruitment, retention and adherence rates are presented with descriptive statistics. Mixed model ANOVAs will be used to determine within-group differences for continuous outcomes between baseline and 3 months, and ANCOVAs for between-group differences for continuous outcomes with baseline score as a co-variate. Intention-to-treat analysis will be used.

# Adverse Events

The Investigator is responsible for the detection and documentation of events meeting the criteria and definitions detailed below.

Participants should be instructed to contact their Investigator and/or treating acupuncturist at any time after consenting to join the trial if any symptoms develop. Treating acupuncturists will enquire about adverse events at every acupuncture visit. All reported Adverse Events (AEs) that occur between consent and the last visit for the study must be recorded in detail in the AE form. In the case of an AE, the Investigator should initiate the appropriate treatment according to their medical judgment. Participants with AEs present at the last visit must be followed up until resolution of the event.

## Definitions

An **Adverse Event** (AE) is any untoward medical event affecting a clinical trial participant. An AE can be any unfavourable and unintended sign (including an abnormal laboratory finding), symptom, or disease (new or exacerbated) temporally associated with the use of a medicinal product, whether or not considered related to the intervention. Each initial AE will be considered for severity, causality or expectedness by the Investigator and may be reclassified as a serious event or reaction based on prevailing circumstances.

An **Adverse Reaction** (AR) is where it is suspected that an AE has been caused by a reaction to acupuncture

A **Serious Adverse Event** (SAE) is any untoward event that occurs at any dose which:

- results in death;
- is life threatening (i.e. the subject was at risk of death at the time of the event; it does not refer to an event which hypothetically might have caused death if it were more severe);
- requires hospitalisation or prolongation of existing hospitalisation;
- results in persistent or significant disability or incapacity;
- is a congenital anomaly or birth defect and/or;
- is considered serious by the Investigator.

Note: Hospitalisations for treatment planned prior to randomisation and hospitalisation for elective treatment of a pre-existing condition will not be considered as an AE. Complications occurring during such hospitalisation will be AEs.

A **Serious Adverse Reaction** (SAR) is an SAE which is causally related to acupuncture. A **Suspected Unexpected Serious Adverse Reaction** (SUSAR) is any SAE which is and which is suspected to be related to the Investigational Product.

## Detecting AEs and SAEs

All AEs and SAEs must be recorded from the time a participant consents to join the study until the last study visit.

The Investigator, treating acupuncturist and research assistant will monitor each subject for Adverse Events during the study. All Adverse Events reported between consent and final follow-up will be recorded in the AE form. The Investigator or designee will ask the subject non-leading questions in an effort to detect Adverse Events e.g.:

“How are you feeling?”

or

“Since you were last asked, have you felt unwell or different from usual?”

In addition, subjects should be encouraged to spontaneously report any unusual feelings or sensations.

Participants should also be asked if they have been admitted to hospital, had any accidents, used any new medicines or changed concomitant medication regimens. If there is any doubt as to whether a clinical observation is an AE, the event should be recorded.

## Managing potential risks

Most serious adverse events that arise from acupuncture treatment will be avoided in this trial due to the application of exclusion criteria and the choice of acupoint location and type of needles. Pneumothorax and cardiac tamponade are highly unlikely as no points on the thorax will be used. Hepatitis and other blood borne viruses are unlikely to be contracted by the participant as only sterile disposable needles will be used. Compartment syndrome is unlikely as no anti-coagulated participants will be accepted into the trial. Endocarditis occurs in participants with prosthetic valves, and these participants are excluded from the trial. Neurological damage and deep venous thrombosis are possible, though rare, and measures to avoid these during the trial include careful acupoint location and avoidance of vigorous stimulation of the needle.

A systematic review of adverse events in auricular acupuncture trials found relatively few reports of adverse events, and no reports of serious adverse events. The adverse events for auricular acupuncture (total of 1753 participants monitored) included:

- pain and tenderness (7.6%)
- minor bleeding (1.4%) which resolved immediately with pressure
- Dizziness (2.9%) which resolved spontaneously
- mild inflammation (0.1%)
- discomfort (1.4%)

Adverse events for auricular electro-acupuncture (total of 203 participants monitored) included:

- discomfort (1%)
- pain (0.5%)

In our partner's experience with the Neurova during an uncontrolled clinical trial (16 participants) there were no adverse events relating to pain, inflammation or bleeding. One participant experienced discomfort.

There have been seven published case reports of perichondritis after auricular acupuncture {Baltimore, 1976 #4216;Davis, 1985 #4214;Gilbert, 1987 #4212;Johansen, 1990 #4211;Sorensen, 1990 #4210}. All occurred before 1990. The lack of reports of perichondritis in the past 27 years may represent increased awareness of the importance of adequate precautions to minimise the risk of infection. In this trial, the concha of the ear is to be cleaned thoroughly with an alcohol swab prior to insertion of the sterile needles. Perpendicular insertion into the cartilage of the ear is to be avoided, instead the insertion of the small needles is safest when done obliquely. Participants are instructed to seek medical advice immediately upon any signs of infection such as redness, pain, heat, purulence or swelling.

The auricular ear-acupuncture stimulation device has not been trialled in pregnancy. Due to this lack of safety data, any woman intending on falling pregnant during the intervention period is to be excluded. Women are asked to avoid falling pregnant, and cease using the ear acupuncture device should they become pregnant.

## Managing adverse outcomes

Participants will be informed of the possible adverse outcomes arising from acupuncture treatment, including the “minor” outcomes such as fainting and pain around needle insertion sites. Practitioners will receive “refresher” training on dealing with common adverse events during treatment, and information on prevention and treatment of adverse events are also found in the Practitioner Training Manual.

Any adverse outcomes occurring during treatment will be dealt with as appropriate by the treating practitioner as below:

- Bleeding or bruising: gentle, firm pressure is applied with a cotton ball for at least 5 minutes. If bruising is large, a cold pack may be applied.
- Drowsiness: participants are to remain in treatment or waiting room until drowsiness passes, unless they are being driven home by somebody else.
- Pain during treatment – The needle is withdrawn slightly if pain persists; if pain does not improve and the participant is too uncomfortable, the needle is withdrawn completely.
- Pain after needle is removed – if there is no bruise/haematoma, the area is to be gently massaged.
- Syncope (fainting) – all needles are removed; participants to be laid flat (no pillow under the head) and with raised legs. Breathing and pulse rate are to be assessed every two minutes. If the situation is normal or improving, the participant is allowed to rest or recover. If the situation deteriorates, strategies such as massaging GV20 and letting other people know the situation and asking for their help. An ambulance should be called if needed.
- Bent needle: the needle is withdrawn slowly if possible (participant may need to return to original position)
- Needle breakage: the needle is removed with a pair of artery forceps if the residual needle is above the skin. If the needle is below skin level, the practitioner will mark and stabilise the area and refer to a hospital or GP for surgical removal. Dizziness: the participant is allowed to lie down until dizziness passes.
- Fatigue after acupuncture: gentler manipulation will be used for the next session.

## Recording AEs AND SAEs

Depending on severity, when an AE/SAE occurs, it is the responsibility of the Investigator to review all documentation related to the event. The Investigator should then record all relevant information in the AE form.

Information to be collected includes dose, type of event, onset date, Investigator assessment of severity and causality, date of resolution as well as treatment required, investigations needed and outcome.

## Evaluation OF AEs AND SAEs

Seriousness, causality, severity and expectedness should be evaluated as though the participant is taking active drug. Cases that are considered serious, possibly, probably or definitely related to drug and unexpected (i.e. SUSARs) are likely to be unblinded.

### Assessment of Seriousness

The Investigator should make an assessment of seriousness as defined in 10.1.

### Assessment of Causality

The Investigator must make an assessment of whether the AE/SAE is likely to be related to treatment according to the following definitions:

**Unrelated**: where an event is not considered to be related to the study drug.

**Possibly**: although a relationship to the study drug cannot be completely ruled out, the nature of the event, the underlying disease, concomitant medication or temporal relationship make other explanations possible.

**Probably**: the temporal relationship and absence of a more likely explanation suggest the event could be related to the study drug.

**Definitely**: The known effects of the study drug or its therapeutic class, or based on challenge testing, suggest that study drug is the most likely cause.

All AEs/SAEs judged as having a reasonable suspected causal relationship (e.g. possibly, probably, definitely) to the study drug will be considered as ARs/SARs. All AEs/SAEs judged as being related (e.g. possibly, probably, definitely) to an interaction between the study drug and another drug will also be considered to be ARs/SAR.

Alternative causes such as natural history of the underlying disease, concomitant therapy, other risk factors and the temporal relationship of the event to the treatment should be considered. The blind should not be broken for the purpose of making this assessment.

### Assessment of Severity

The Investigator should make an assessment of severity for each AE/SAE and record this on the CRF according to one of the following categories:

**Mild**: an event that is easily tolerated by the participant, causing minimal discomfort and not interfering with every day activities.

**Moderate**: an event that is sufficiently discomforting to interfere with normal everyday activities.

**Severe**: an event that prevents normal everyday activities.

Note: the term ‘severe’, used to describe the intensity, should not be confused with ‘serious’ which is a regulatory definition based on participant/event outcome or action criteria. For example, a headache may be severe but not serious, while a minor stroke is serious but may not be severe.

### Assessment of Expectedness

If an event is judged to be an AR/SAR, the evaluation of expectedness should be made based on knowledge of the reaction.

## Reporting of SAEs/SARs/SUSARs

Once the Investigator becomes aware that an SAE has occurred in a study participant, they must report the information to the Sponsor within 24 hours of becoming aware of the event. The SAE form must be completed as thoroughly as possible with all available details of the event, signed by the Investigator or designee. If all the required information is not available at the time of reporting, the Investigator must ensure that any missing information is provided as soon as this becomes available. It should be indicated on the report that this information is follow-up information of a previously reported event.

The SAE report must provide an assessment of causality and expectedness at the time of the initial report to the Sponsor according to Sections 10.4.2 Assessment of Causality and 10.4.4 Assessment of Expectedness.

## Follow up Procedures

After initially recording an AE or recording and reporting an SAE, the Investigator is required to follow each participant until the AE/SAE resolves or is considered stable by both the Investigator and Sponsor if ongoing at the final follow-up visit. Follow up information for an SAE should be reported to the Sponsor.

# Good Clinical Practice Module

## Ethical Conduct of the Study

This study will be carried out according to the Declaration of Helsinki, the NHMRC National Statement on Ethical Conduct in Research Involving Humans (1999) and the Notes for Guidance on Good Clinical Practice as adopted by the Australian Therapeutic Goods Administration (2000) (CPMP/ICH/135/95) and the ICH GCP Guidelines.

The protocol and related documents will be submitted for review by the Human Research Ethics Committee (HREC) and written approval received before the study can commence.

## Investigator Responsibilities

The Investigator is responsible for the overall conduct of the study at the site and compliance with the protocol and any protocol amendments. In accordance with the principles of GCP, the following areas listed in this section are also the responsibility of the Investigator. Responsibilities may be delegated to an appropriate member of study site staff. Delegated tasks must be documented on a Delegation Log and signed by all those named on the list.

## Informed Consent

The Investigator will obtain written informed consent from each participant prior to participation in the study, in accordance with International Conference on Harmonisation of Technical Requirements for Registration of Pharmaceuticals for Human Use (ICH) Good Clinical Practice (GCP) Guidelines, Declaration of Helsinki 2000, and any local regulatory requirements

The decision of a participant to participate in clinical research is voluntary and should be based on a clear understanding of what is involved.

Participants will receive adequate oral and written information; Participant Information Sheet and Informed Consent Forms will be provided. The oral explanation to the participant should be performed by the Investigator or designated person, and will cover all the elements specified in the Participant Information Sheet/Informed Consent.

The participant must be given sufficient time to consider the information provided. The participant may withdraw their consent to participate at any time without loss of benefits to which they otherwise would be entitled.

The Investigator or delegated member of the trial team and the participant will sign and date the Informed Consent Form(s) to confirm that consent has been obtained. The participant will receive a copy of this document and a copy filed in the Site Master File (SMF).

## Emergency Contact with Investigators

All subjects will be provided with a Subject Emergency Contact Card with contact details of whom to contact in the case of an emergency.

## Notification of Primary Care Physician

With the consent of the volunteer, it is the Investigator’s responsibility to notify the primary care physician of the participant’s participation in the study, provided that such a physician can be identified and this notification is relevant for the particular circumstances of the trial. Where relevant, a letter will be sent to the physician stating the nature of the study, treatments, expected benefits or Adverse Events and concomitant drugs to be avoided. A copy shall be retained by the study site for verification by the Study Monitor.

## Investigator Indemnification

Western Sydney University will reimburse subjects for costs of medical care that occur as a result of complications directly related to participation in this study.

## Study Site Staff

The Investigator must be familiar with protocol and the study requirements. It is the Investigator’s responsibility to ensure that all staff assisting with the study are adequately informed about the Investigational Product, protocol and their trial related duties.

## Data Recording

The Investigator is responsible for the quality of the data recorded in the CRF.

### Confidentiality

All, evaluation forms, reports, and other records must be identified in a manner designed to maintain participant confidentiality. All records must be kept in a secure storage area with limited access. Clinical information will not be released without the written permission of the participant, except as necessary for monitoring and auditing by the Sponsor, its designee, Regulatory Authorities, or the HREC. The Investigator and study site staff involved with this study may not disclose or use for any purpose other than performance of the study, any data, record, or other unpublished, confidential information disclosed to those individuals for the purpose of the study. Prior written agreement from the Sponsor or its designee must be obtained for the disclosure of any said confidential information to other parties.

### Data Protection

All Investigators and study site staff involved with this study must comply with the requirements of the appropriate Data Protection or Privacy Act with regard to the collection, storage, processing and disclosure of personal information and will uphold the Act’s core principles. Access to collated participant data will be restricted to those clinicians treating the participants.

Computers used to collate the data will have limited access measures via user names and passwords.

The only data that will contain identifiers (Name, DOB, Contact details) is the pathology that is collected by Laverty Pathology, and a tracking spreadsheet that the research team uses to keep track of participant progress through the study. Biological samples are labeled with participant name DOB and processed according to standard Laverty Pathology operating procedures which include access only by authorized personnel. Pathology results are uploaded onto Laverty Pathology’s online portal Medway. This portal requires password access and contains an audit trail. Pathology data from the Laverty portal will then be entered into Redcap by an authorized research assistant. No identifying details will be entered into Redcap. Any pathology reports that are received in paper format are de-identified (identified by ID number only) and stored securely in Dr Carolyn Ee’s locked filing cabinet or in a secure password protected folder on Owncloud. The tracking spreadsheet is password protected and stored on a password protected folder on Owncloud and is accessible only to the research team that is involved in recruitment and screening. It is stored away from any medical details about participants.

if clinically approved tests are conducted (e.g., blood counts), then these results will be provided to the treating physician within weeks, unless "panic values" are found, in which case results will be reported to the physician and study subject within 24-48 hours.

If there is email communication about participants that contains medical and other personal information, only ID numbers are used.

Published results will not contain any personal data that could allow identification of individual participants.

# Study Administrative Procedures

## Protocol Amendments

Any changes in research activity, except those necessary to remove an apparent, immediate hazard to the participant, must be reviewed and approved by the Chief Investigator. Amendments to the protocol must be submitted in writing to the appropriate HREC, for approval prior to participants being enrolled into an amended protocol.

## Protocol Violations and Deviations

The Investigator should not implement any deviation from the protocol without agreement from the Chief Investigator and appropriate HREC approval except where necessary to eliminate an immediate hazard to trial participants.

In the event that an Investigator needs to deviate from the protocol, the nature of and reasons for the deviation should be recorded in the CRF. If this necessitates a subsequent protocol amendment, this should be submitted to the HREC for review and approval if appropriate.

## Study Record Retention

Study records will be retained for a minimum of 5 years. Participant files and other essential documents (study protocol, Site Master File (SMF) signed Informed Consent Formscorrespondence, CRFs, source documents and other documents pertaining to the conduct of the study) must be kept for the maximum period permitted by the research institute in accordance with these requirements.

Should the Investigator wish to assign the study documentation to another party or move to another location, the trial Coordinating Centre / Study Sponsor should be notified.

## End of Study

The end of study is defined as the last participant’s last visit.

The Investigators and/or the trial steering committee have the right at any time to terminate the study for clinical or administrative reasons.

A summary report of the study will be provided to the HREC at the end of the study.

# Reporting, Publications and Notification of Results

## Authorship Policy

Ownership of the data arising from this study resides with the study team. On completion of the study, the study data will be analysed and tabulated, and a clinical study report will be prepared.

## Publication

The clinical study report will be used for publication and presentation at scientific meetings. Investigators have the right to publish orally or in writing the results of the study.

Summaries of results will also be made available to Investigators for dissemination within their clinics (where appropriate and according to their discretion).

# Appendix 1 - Schedule of Observations and Procedures

| **Visit/Timepoint** | **Screening (self screening or telephone)** | **Week -1**  **(Run-in)** | **Visit 1**  **Baseline (Allocation)** | **1^st^ acupuncture treatment** | **Visit 2**  **Post intervention** |
| --- | --- | --- | --- | --- | --- |
| Week(s): | **-4 to -2** | **-1** | **0** | **1** | **12** |
| **ENROLMENT** |  |  |  |  |  |
| Eligibility screen | **X** |  |  |  |  |
| Informed Consent |  |  | **X** |  |  |
| 2hr OGTT and Insulin, free and total testosterone, SHBG, Free androgen index |  | **x** |  |  | **x** |
| Physical activity levels |  | **x** |  |  | **X (Week 11)** |
| Allocation |  |  | **X** |  |  |
| **INTERVENTIONS** |  |  |  |  |  |
| Body acupuncture + lifestyle |  |  |  |  |  |
| Auricular electro acupuncture + lifestyle |  |  |  |  |  |
| Lifestyle alone |  |  |  |  |  |
| **ASSESSMENTS** |  |  |  |  |  |
| Credibility and Expectancy Questionnaire |  |  |  | **X** |  |
| Demographic and medical questionnaire |  |  | **X** |  |  |
| Anthropometric measures and blood pressure |  |  | **X** |  | **X** |
| Concomitant Medications |  |  | **X** |  | **X** |
| Surveys – QoL, psychological, weight self efficacy, locus of control |  |  | **X** |  | **X** |
| Heart rate variability by ECG monitor |  |  | **x** |  | **x** |
| Ferriman-Gallwey score |  |  | **X** |  | **X** |
| Exit survey on acceptability of the intervention |  |  |  |  | **X** |
| Adverse Events |  |  | **X** | **X** | **X** |

# References

1. Teede, H., A. Deeks, and L. Moran, *Polycystic ovary syndrome: a complex condition with psychological, reproductive and metabolic manifestations that impacts on health across the lifespan.* BMC Med, 2010. **8**: p. 41.

2. Moran, L.J., et al., *Polycystic ovary syndrome and weight management.* Womens Health (Lond Engl), 2010. **6**(2): p. 271-83.

3. Jean Hailes Foundation for Women's Health on behalf of the PCOS Australian Alliance, *Evidence-based guideline for the assessment and management of polycystic ovary syndrome*. 2011: Melbourne.

4. Abdi, H., et al., *The effects of body acupuncture on obesity: anthropometric parameters, lipid profile, and inflammatory and immunologic markers.* ScientificWorldJournal, 2012. **2012**: p. 603539.

5. Gucel, F., et al., *Influence of acupuncture on leptin, ghrelin, insulin and cholecystokinin in obese women: a randomised, sham-controlled preliminary trial.* Acupunct Med, 2012. **30**(3): p. 203-7.

6. Schukro, R.P., et al., *The effects of auricular electroacupuncture on obesity in female patients--a prospective randomized placebo-controlled pilot study.* Complement Ther Med, 2014. **22**(1): p. 21-5.

7. Firouzjaei, A., et al., *Comparative evaluation of the therapeutic effect of metformin monotherapy with metformin and acupuncture combined therapy on weight loss and insulin sensitivity in diabetic patients.* Nutr Diabetes, 2016. **6**: p. e209.

8. Belivani, M., et al., *Acupuncture in the treatment of obesity: a narrative review of the literature.* Acupunct Med, 2013. **31**(1): p. 88-97.

9. Ren, L.N., et al., *[A meta-analysis on acupuncture treatment of polycystic ovary syndrome].* Zhen Ci Yan Jiu, 2014. **39**(3): p. 238-46.

10. Qu, F., et al., *The effects of acupuncture on polycystic ovary syndrome: A systematic review and meta-analysis.* European Journal of Integrative Medicine, 2016. **8**(1): p. 12-18.

11. Witt, C.M., et al., *Safety of Acupuncture: Results of a Prospective Observational Study with 229,230 Patients and Introduction of a Medical Information and Consent Form.* Forschende Komplementarmedizin, 2009. **16**(2): p. 91-97.

12. Sully, B.G., S.A. Julious, and J. Nicholl, *A reinvestigation of recruitment to randomised, controlled, multicenter trials: a review of trials funded by two UK funding agencies.* Trials, 2013. **14**: p. 166.

13. O'Cathain, A., et al., *Maximising the impact of qualitative research in feasibility studies for randomised controlled trials: guidance for researchers.* Trials, 2015. **16**(Suppl 2): p. O88-O88.

14. Teede, H.J., et al., *Assessment and management of polycystic ovary syndrome: summary of an evidence-based guideline.* Med J Aust, 2011. **195**(6): p. S65-112.

15. Richards, D. and J. Marley, *Stimulation of auricular acupuncture points in weight loss.* Aust Fam Physician, 1998. **27 Suppl 2**: p. S73-7.

16. Kuang, H., et al., *Acupuncture and clomiphene citrate for live birth in polycystic ovary syndrome: study design of a randomized controlled trial.* Evid Based Complement Alternat Med, 2013. **2013**: p. 527303.

17. White, A.R., J. Filshie, and T.M. Cummings, *Clinical trials of acupuncture: consensus recommendations for optimal treatment, sham controls and blinding.* Complement Ther Med, 2001. **9**(4): p. 237-45.

18. Cabioglu, M.T. and N. Ergene, *Changes in levels of serum insulin, C-Peptide and glucose after electroacupuncture and diet therapy in obese women.* Am J Chin Med, 2006. **34**(3): p. 367-76.

19. O'Hara, B., et al., *The NSW Get Healthy Information and Coaching Service: the first five years.* . 2014, NSW Ministry of Health & Prevention Research Collaboration, University of Sydney: NSW.
