# Supplementary material for: Acupuncture or auricular electro-acupuncture as adjuncts to lifestyle interventions for weight management in PCOS: protocol for a randomised controlled feasibility study
Source: Pilot Feasibility Stud. 2020 Apr 25;6:53. doi: 10.1186/s40814-020-00591-4 (PMC7183107; doi:10.1186/s40814-020-00591-4)
Supplement: Supplementary file 1 — Additional file 1. Acupuncture for PCOS protocol R1. SPIRIT FigureR1. [file 40814_2020_591_MOESM1_ESM.zip › Appendix 2 SPIRIT FigureR1.docx]

**Appendix 2 SPIRIT Figure**

| **Visit/Timepoint** | **Screening (self screening or telephone)** | **Week -1**  **(Run-in)** | **Visit 1**  **Baseline (Allocation)** | **1^st^ acupuncture treatment** | **Visit 2**  **Post intervention** |
| --- | --- | --- | --- | --- | --- |
| Week(s): | **-4 to -2** | **-1** | **0** | **1** | **12** |
| **ENROLMENT** |  |  |  |  |  |
| Eligibility screen | **X** |  |  |  |  |
| Informed Consent |  |  | **X** |  |  |
| 2hr OGTT and Insulin, free and total testosterone, SHBG, Free androgen index |  | **x** |  |  | **x** |
| Physical activity levels |  | **x** |  |  | **X (Week 11)** |
| Allocation |  |  | **X** |  |  |
| **INTERVENTIONS** |  |  |  |  |  |
| Body acupuncture + lifestyle |  |  |  |  |  |
| Auricular electro acupuncture + lifestyle |  |  |  |  |  |
| Lifestyle alone |  |  |  |  |  |
| **ASSESSMENTS** |  |  |  |  |  |
| Credibility and Expectancy Questionnaire |  |  |  | **X** |  |
| Demographic and medical questionnaire |  |  | **X** |  |  |
| Anthropometric measures and blood pressure |  |  | **X** |  | **X** |
| Concomitant Medications |  |  | **X** |  | **X** |
| Surveys – QoL, psychological, weight self efficacy, locus of control |  |  | **X** |  | **X** |
| Heart rate variability by ECG monitor |  |  | **x** |  | **x** |
| Ferriman-Gallwey score |  |  | **X** |  | **X** |
| Exit survey on acceptability of the intervention |  |  |  |  | **X** |
| Adverse Events |  |  | **X** | **X** | **X** |
